# Seam Properties of Overlap Welding Strategies from Copper to Aluminum Using Green Laser Radiation for Battery Tab Connections in Electric Vehicles

**DOI:** 10.3390/ma16031069

**Published:** 2023-01-25

**Authors:** Florian Kaufmann, Mihail Strugulea, Christian Höltgen, Stephan Roth, Michael Schmidt

**Affiliations:** 1 Bayerisches Laserzentrum GmbH (blz), Konrad-Zuse-Str. 2-6, 91052 Erlangen, Germany; 2 Institute of Photonic Technologies (LPT), Friedrich-Alexander Universität Erlangen-Nürnberg, Konrad Zuse-Str. 3-5, 91052 Erlangen, Germany; 3 Chair of Production Engineering and Innovation Management, Werkzeugmaschinenlabor (WZL) of RWTH Aachen University, Campus Boulevard 30, 52074 Aachen, Germany; 4 Erlangen Graduate School in Advanced Optical Technologies (SAOT), Paul-Gordan-Str. 6, 91052 Erlangen, Germany

**Keywords:** electromobility, visible laser radiation, laser beam micro welding, dissimilar metals welding, spatial power modulation, battery tab connection, intermetallic phases

## Abstract

Laser beam welding of metals has progressed dramatically over the last years mainly arising from joining applications in the field of electromobility. Allowing the flexible, automated manufacturing of mechanically, electrically, and thermally stressed components, the process is more frequently applied for joining highly reflective materials, for example for battery tab and busbar connections. The local, non-contact energy input favors this welding technology; however, joining of copper and aluminum sheets still poses a challenge due to the physical properties of the joining partners and intermetallic phases from dissimilar metal interaction, which reduce seam performance. The use of green laser radiation compared to infrared laser radiation offers the advantage of a significantly increased absorptivity for copper materials. A changed incoupling behavior is observed, and a lower deep penetration threshold has been already proven for 515 nm wavelength. When copper and aluminum are welded with the former as top sheet, this welding mode is essential to overcome limited aspect ratios from heat conduction welding. However, the opportunities of applying these beam sources in combination with spatial power modulation to influence the interconnection area of copper-aluminum joints have not yet been studied. The aim of this work is therefore to investigate the seam properties and process stability of different overlap welding strategies using green laser radiation for dissimilar metal welding. A microstructural analysis of the different fusion zones and mechanical strength of the joints are presented. In addition, the experimental parameter sets were analyzed regarding their application in battery module busbars by examining the electrical resistance and temperature distribution after welding. A parameter window was identified for all investigated welding strategies, with the stitched seam achieving the most stable results.

## 1. Introduction

Road transportation is responsible for about one -fifth of the greenhouse gas emissions in the EU-27 states, with passenger vehicles making the largest contribution to this sector with 61% [1]. Considering that the share has remained largely constant throughout recent years, the electrification of vehicles in both the individual and shared mobility is the only viable option to reduce CO_2_ emissions to the targeted level of less than 80.8 g CO_2_/km by 2025 [2].

In this context, the importance of sustainable drive concepts has increased and a transformation from vehicles with internal combustion engines to electric vehicles has begun in recent years [3]. In addition, the use of electronic comfort functions in internal combustion engine vehicles has increased rapidly with the emergence of electric vehicles. These developments require energy storage systems on an unknown scale, which are therefore the key factor for this progression [4]. Due to their high energy density, lithium-ion batteries are currently used as accumulators in electric vehicles and hybrid electrical vehicles (including plug-in hybrid electric vehicles) [5]. Depending on the area of application, different battery cell variants are used that have been technically adapted to the respective application [6]. Existing variants of lithium-ion batteries differ in the housing type [7], the electrode geometries [8], and the chemical composition of the active layers [9]. The electrodes of these batteries commonly consist of aluminum, due to its easy processability, and copper, which has high electrical conductivity [10]. When connecting more cells to a battery module, aluminum and copper have to be joined together. Considering pouch cells due to their high volumetric energy density, this implies the joining task of sub-mm thick battery tab connectors, also called arrester tabs [11].

A second major challenge arising from the additional weight of the battery packs compared to internal combustion engines is the need for lightweight constructions. Mass reduction for automotive applications is considered one of the most promising approaches to reduce energy consumption, focusing on the use of ferrous and non-ferrous metals as well as fiber-reinforced materials in combination for structural components. This multi-material mixture requires the joining of different materials with different strength, deformability, melting points, and heat conductivity. However, the mass reduction also involves wiring and cable systems, as the total mass in cars can reach between 15 and 45 kg. By a reduction of the cross section at a comparable resistivity or by a substitution of copper with lighter aluminum, a mass reduction of up to 40% can be achieved. A comparable resistivity of a copper wire (conductivity σ = 58 m/(Ω mm^2^; density δ = 8.93 g/mm^3^) at a diameter of 10 mm can be achieved with an aluminum wire of 16 mm diameter (σ = 35.5 m/(Ω mm^2^; δ = 2.70 g/mm^3^) [12].

**Figure 1:**
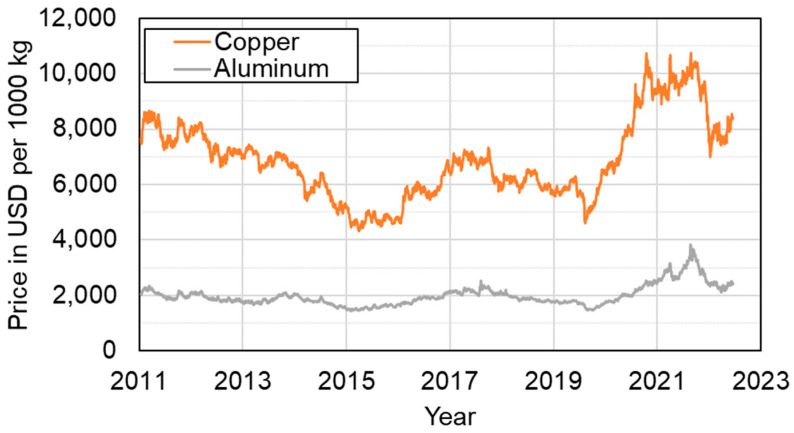
Price development of copper and aluminum in US dollar, data extracted from [13].

In addition, the raw material price of aluminum is more stable and is only about a quarter of the price for copper (see Figure 1), so long-term economic planning is safer. This method has its limitations, as larger cross-sections also require more space thus reducing driver comfort.

In summary, the main drivers for dissimilar copper to aluminum connections can therefore be found in battery tab connections as well as in wiring/electrical systems. The connection of copper to aluminum can be achieved through mechanical fastening or crimping. Thereby a form locking is achieved, which can deteriorate due to relaxation or partly releasing. The mechanical joining processes also prove to be strongly dependent on environmental conditions and humidity in terms of their contact resistance. This results in a high amount of losses in the wiring system or in the battery power system [14].

The joining of copper to aluminum forming a bonding with metal continuity also proves to be advantageous in long-term applications. Solid-state welding methods such as ultrasonic welding or friction stir welding obtain good results concerning a limitation of intermetallic phases in the weld seam due to their low joining temperatures [15]. However, limitations such as restrictions on joint types, a large weld indentation, and the limited accessibility of the joining partners can restrict their application. Within the scope of this work, laser beam welding was therefore investigated for this demanding application, which is described in detail in the following chapters.

## 2. State of the Art

The joining process used for dissimilar material connection must be designed to provide product quality and production efficiency benefits as well as it must meet the definition of a green low-carbon manufacturing process for battery equipment [16].

### 2.1. Laser Beam Welding and Application of Spatial Power Modulation

The automated production of mechanically, electrically, and thermally loaded components in this sector is therefore more frequently realized using laser beam welding. Especially when joining materials with high heat conductivity, such as copper, the local, non-contact energy input favors this welding technology [17]. According to DIN 1910-100, the process is a subgroup of fusion welding, whose energy carrier is radiation [18].

Depending on the intensity level used, the regimes heat conduction welding or deep penetration welding can be achieved. In the heat conduction welding mode, the absorbed irradiance leads to local melting on the metals surface in the area of the laser spot. The energy is transferred inside the material by heat conduction. Therefore, the resulting weld seams are limited to small ratios of significantly less than one (ratio of penetration depth to seam width) for the single laser-matter interaction [19].

When the intensity in the interaction zone is increased over a material-dependent threshold value, the metal is vaporized, and deep penetration welding occurs. A characteristic feature of deep penetration welding is the formation of a vapor capillary in the material, the so-called keyhole. Starting from the depression of the melt pool surface induced by recoil pressure, multiple reflections within the forming keyhole potentiate the degree of energy coupling into the material. As a result, deep and narrow laser-matter interaction areas (high aspect ratios, up to 10:1) can be achieved compared to heat conduction welding. By continuous movement of the beam, the solidifying melt forms a weld seam behind the irradiated zone [20]. However, a technical zero gap is necessary for both regimes to create a sufficient material bond between two joining partners. Important input parameters of the welding process are the laser spot characteristics and the physical properties of the materials as well as the process parameters laser power, feed rate, and seam geometry [21].

As especially due to the increased dynamics in deep penetration welding, the occurrence of process instabilities is detrimental, special welding strategies are used to reduce fluctuations. The flexibility of the process allows the laser spot trajectory to be easily adjusted. Through the implementation of spatial power modulation, the weld seam width can be increased and nearly rectangular cross sections are achievable [22]. This strategy is accompanied by a smaller weld seam depth as the local path speed is increased, but at the same time a reduction in seam imperfection amount was observed. The laser beam manipulation differs from the conventional strategy by superposing an oscillation movement such as a circular or eight (infinite) motion to the global linear feed rate. The so-called Lissajous figures provide the basis for mapping the oscillation trajectory when superimposing a harmonic oscillation with constant amplitude in one or two coordinate directions [23]. The beam trajectory is defined by different degrees of overlap depending on the oscillation parameters and feed rate selected.

Investigations using spatial power modulation also revealed an increased efficiency of the welding process [24]. Comparing spatial power modulation with circular oscillation and a linear weld at constant linear feed rate and laser power, the overall cross-sectional area is larger in the former case [23]. This increase in weld seam width may be particularly advantageous for overlap joints to reduce the electrical contact resistance.

### 2.2. Laser-Based Overlap Joining of Dissimilar Metals

Basically, welding dissimilar metals such as copper and aluminum are more complex than joining metals of the same material group [25]. This can be attributed to different physical and metallurgical properties of the joining partners, thus influencing the welding process in different manners. One challenge is the difference in the melting temperature of copper and aluminum, which is more than 400 K (T_M,Cu_ = 1083 °C, T_M,Al_ = 660 °C, [26]). This fact leads to additional temperature induced dynamics in the melt pool due to the temperature gradient. Furthermore, in situ synchrotron investigations revealed different melt pool extensions, whereby uprising bubbles were trapped in the area of the material transition and remained as pores in the joint [27]. Different coefficients of thermal expansion (α_Cu_ = 17 × 10^−6^∙K^−1^, α_Al_ = 24 × 10^−6^∙K^−1^, [26]) promote stresses between the joining partners, especially during cooling. The different solidification rates can cause stresses in the weld seam which can lead to cracks [28].

Especially when joining aluminum with steel [29] and copper with aluminum [10], the limited solubility is the root cause for the formation of intermetallic phases in the common melt pool during welding. Depending on the mixing ratio, different intermetallic phases are formed, which have a significantly higher hardness, can lead to brittle fracture in the joined area under mechanical load, and increase the electrical resistance respectively [30]. Table 1 shows the most important intermetallic phases formed when welding copper and aluminum and their physical properties. It becomes clear that Al_2_Cu has the lowest activation energy and Al_4_Cu_9_ has the highest [31].

The specific resistivity increases significantly for all phases compared to pure copper and shows the highest value of 14.2 µΩ cm for the γ_1_-phase. In terms of mechanical properties, the hardness increases with increasing copper content, with the exception of the δ-phase [33]. Braunovic et al. showed a linear relationship between the thickness of the intermetallic phase layer and its electrical resistivity. In addition, it was found that the welded joint becomes very brittle at a intermetallic layer thickness above 5 μm [34]. This embrittlement of the joint may also have a negative influence on the tensile strength. Since these intermetallic phases reduce the weld seam performance, but their formation is inevitable in a laser welding process, the general aim is to reduce their amount to the possible minimum. Therefore, different approaches are being pursued in literature.

On the one hand, the presence of a keyhole in deep penetration welding mode supports metallurgical mixing in the melt pool, see [35,36]. By optimizing the process parameters such as feed rate, laser power, and weld geometry, this mixing in the joining zone can be controlled accordingly. Xue et al. [37] studied the linear overlap welding of 0.3 mm thick copper and aluminum by means of experimental investigations and simulation. The interdiffusion of copper and aluminum in the molten state was found to be extensive when copper was used as top plate. The joint was found to be full of various intermetallic compounds. The intermediate layer had an arch shape because the fluid flows from aluminum to copper near the interface in the weld pool. When aluminum was placed on top of copper, the interdiffusion of copper and aluminum was found to be limited by a small amount of copper melting. The weld pool had a characteristic bowl-shape with the fusion zone being wider at the top of both materials. This behavior is assumed to be caused by convective mixing and a lower melting temperature of the mixture [37]. In addition, a lateral beam offset proved to be comparatively advantageous in butt weld configuration [38]. Special laser beam trajectories have also been subjected to experimental investigations [16].

Leera et al. [39] investigated the influence of pulse shape and pulse to pulse distance in pulsed laser beam welding. Improved results in terms of mechanical strength and electrical resistance were obtained when pulse shapes adopting preheating were used. The authors in [40] developed a process control by real-time pulse shaping with a control loop smaller than 10 µs. By adjusting the laser power in each individual pulse after detecting the material specific process emissions, the laser beam welding of different metals with a nearly constant penetration depth at the interface was demonstrated. Furthermore, investigations revealed that intermediate layers of silver, zinc, or tin may effectively reduce the formation of intermetallic phases. A positive effect regarding the tensile strength was found in the work of Mys et al. [41].

Compared to conventional laser beam welding, spatial power modulation with circular oscillation movement reduces the solidification rate due an overlapping laser trajectory. According to the authors in [42], this reduces the detrimental phase formation as well as the residual stresses and associated deformations. In [35], the material mixing in the fused zone was found enhanced and the formation of cracks along the contact area of both sheets was reduced for larger scanning amplitudes. Since the locally introduced laser energy per unit length decreases with both parameters, the oscillations amplitude and the frequency, the obtained weld depth decreased with both parameters. Thus, it was shown that the average relative share of copper in the weld seam could to be influenced in the range of 0 to 53 wt. % by a proper adjustment of the oscillation parameters [35]. Similarly, Dimatteo et al. investigated a circular laser beam oscillation strategy, achieving good mechanical properties and low electrical resistance with double welds [43].

### 2.3. Influence of Processing Wavelength—Application of Visible Laser Radiation

For the most common laser beam welding processes, near-infrared laser radiation (λ ≈ 1 µm) in continuous wave (cw) mode is employed. The high focusability of the available disk and fiber lasers enables the high intensity required for the keyhole welding process. This welding regime is beneficial for an efficient weld process due to the increased energy coupling inside the keyhole. When welding copper and aluminum with the former as top sheet, this mode is furthermore essential to overcome the limited aspect ratios in heat conduction welding.

For highly reflective metals such as copper, the absorptivity for infrared laser radiation at room temperature in solid state is only in the single digit range [44]. The use of green laser radiation (λ ≈ 515 nm) compared to infrared laser radiation offers the advantage of a significantly increased absorptivity in both solid and liquid state [19]. A changed incoupling behavior is observed and a lower deep penetration threshold have been already proven for the 515 nm wavelength [45]. Analytical studies in [46] based on the energy balance in combination with thermophysical material properties support the experimental findings and confirm a higher process stability for spot welds on copper with green laser radiation. However, the opportunities of applying these beam sources in combination with spatial power modulation to influence the interconnection area of copper-aluminum joints have not yet been studied in detail [47].

Studies in this direction, using visible wavelengths such as green and blue, are currently less comprehensive in the literature [48]. Mathivanan et al. [49] investigated the mechanical properties of copper aluminum welds in overlap configuration using a beam oscillation in the form of infinite shape with 515 nm laser radiation. A discontinuous weld seam was observed in cross-sectional direction, which was related to the different degree of intermixing. The combination of ductile and brittle intermetallic microstructure was found, resulting in a strong joint with large plastic deformation of the aluminum sheet [49]. The authors in [50] focused on conduction mode welding using a blue laser for welding low-thickness dissimilar materials, namely nickel-coated copper and mild steel, for electric vehicle battery manufacturing. Ascari et al. also welded 0.3 mm thick nickel-plated copper sheets to 0.4 mm thick aluminum sheets by using a 450 nm diode laser, finding a smooth seam with no cracks or pores in the weld. The authors stated a larger process window compared to previous experiments with a near-infrared laser beam, achieving similar tensile strengths as in the earlier studies [48].

From the state -of -the -art and the amount of research on this topic, it can be concluded that a deeper understanding of the influencing factors affecting the welding process is absolutely needed and the top plate configuration selection in copper aluminum joining requires methodology. In summary, only limited research has been conducted to study copper as top plate in copper aluminum mixed joints [49], as for example the investigations in [50,51,52]. In addition, the use of visible laser radiation proves high potential for these limited material thicknesses in deep penetration mode to create a reproducible, low fluctuation joint between the two metals.

The aim of this work is therefore to investigate the seam properties and process stability for different overlap welding strategies using green laser radiation for dissimilar copper to aluminum joints. The laser beam is irradiated from the copper side, to distribute the forming intermetallic phases inside the joint in order to obtain a ductile seam behavior and achieve well -suited electrical properties. A microstructural analysis of the different fusion zones is conducted, and the mechanical strength of the joints is examined. In addition, the experimental parameter sets are analyzed regarding their application in battery tab connections by examining the electrical transition resistance and the temperature distribution after welding.

## 3. Materials and Methods

In this paragraph, the welding setup is introduced first. Then the experimental procedure is described, followed by the analysis approaches for the experimental investigation.

### 3.1. Laser Beam Welding Setup

In this work, laser beam welding of copper (Cu) and aluminum (Al) in overlap configuration was investigated applying different welding strategies. Therefore, a welding setup with green laser radiation was used to join battery tab connectors of pouch-type battery cells. The properties of the laser source and the optical setup are listed in Table 2.

**Figure 2:**
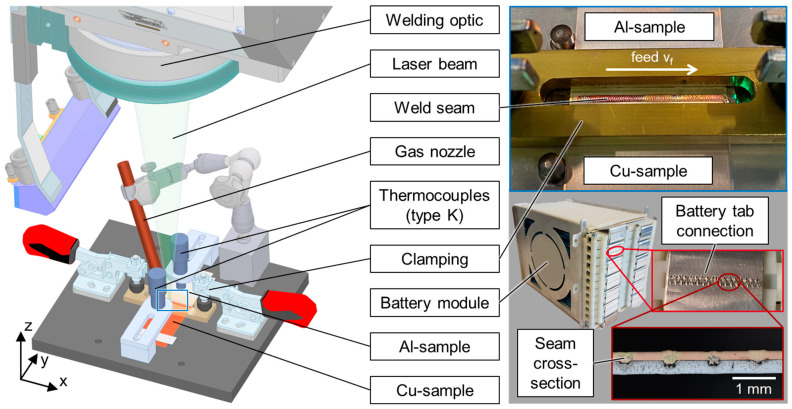
Schematic sketch (CAD-model) of the experimental setup for overlap welding from copper to aluminum, (**left**); detail insert of a welded sample in the real setup (**right**, **top**) and application in battery tab connections for electric vehicles (**right**, **bottom**).

The experiments were performed with a frequency-doubled disk laser TruDisk 3022 (TRUMPF Laser GmbH + Co. KG, Ditzingen, Germany) emitting 3 kW maximum cw-output power at λ = 515 nm wavelength. The laser beam is guided through a fiber-optic cable with a core diameter of d_LLK_ = 200 µm to the welding optics (PFO 33-2). The collimator focal length of f_C_ = 150 mm in combination with a f-theta focusing optics, f_F_ = 255 mm, results in an imaging ratio of 1:1.7. A top hat -shaped intensity profile in the focal plane was measured with a diameter of 342.5 µm using a Primes Focus Monitor FM+. The copper and aluminum connectors were processed in the focal plane at z = 0 mm (copper top surface). The customized experimental setup for the investigation is based on the setup presented in Ref. [52], which was developed in cooperation with the Chair of Production Engineering from the RWTH Aachen University. A schematic sketch of the experimental setup is shown in Figure 2.

For the experimental investigations, an aluminum (Al) and a copper (Cu) sample plate were positioned in overlap configuration in the welding fixture. The dimension of the sheets was 55 mm in length, 35 mm in width, and 0.2 mm thickness for the Cu plate, 0.3 mm for the Al plate respectively. Aluminum AlN30 was used on the one hand and nickel-plated copper plates on the other. The 2.5 μm thick Ni-coating on the Cu-ETP plates was electrodeposited, with a tolerance specified between 0.6 and 3 µm. The chemical composition of the materials is listed in Table 3 according to the supplier inspection sheet from Kunshan Huaiyuan Battery material Co., Ltd.

For sufficient clamping, an overlapping distance of 10 mm was set in the area to be welded. The zero-gap position was achieved using a clamping top beam. The laser beam reached the material at an inclination angle of 90°, as the high absorptivity of copper for green laser radiation does not require tilting the welding optics or working in the outer area of the scan field, and it is typically applied when working with near-infrared lasers for back reflection protection of the system setup. The global feed rate was set in positive x-direction. A welded result can be seen in the detail insert in the top right of Figure 2, which shows a final seam on a clamped sample after welding in the real experimental setup.

For the temperature measurement in the seam adjacent zone, two thermocouples were placed on top of the samples, which were spring-loaded to increase the thermal contact between the workpiece and the sensors. The thermocouples (type K, Therma Thermofühler GmbH, Lindlar, Germany) with 7 mm probe diameter were positioned 20 mm away from the symmetry plane of the weld seam to record the maximum temperatures in the base material during welding. The recording was realized via connection to a data logger (Delphin Expert Key 200L, Delphin Technology AG, Bergisch Gladbach, Germany) with analogue inputs with 18 bit resolution. According to Ref. [53], a maximum permissible temperature of *T_Crit_* = 65 °C should not be exceeded in the seam adjacent zone, since above *T_Crit_*, decomposition reactions in the electrolyte, surrounding the cell-internal battery tab connection, take place. This phenomenon can have a negative impact on the performance and lifetime of the battery cells in the later application of this joining process for cell-external battery tab connections.

Nitrogen was used for processing zone coverage supplied through a lateral gas nozzle to remove the particle plume during welding. In addition, a cross-jet with compressed air protects the welding optics from fume and spatters. The application of the developed welding parameters in a battery module demonstrator can be seen in Figure 2 bottom right, indicating different levels of detail of the investigation.

### 3.2. Overlap Welding Strategies

**Figure 3:**
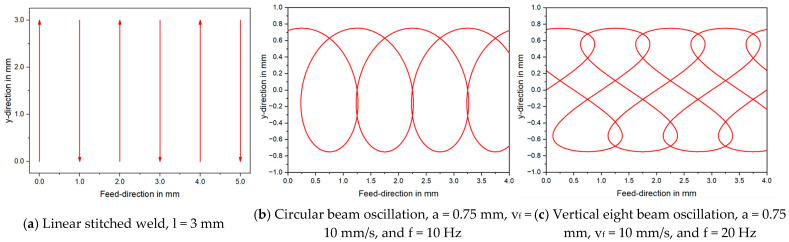
Overlap welding strategies for copper-aluminum welds used in the experimental investigation: linear stitched weld (**a**), circular beam oscillation (**b**), and vertical eight beam oscillation (**c**).

For the experimental investigations, three different welding strategies were examined. These include a linear stitched weld and spatial laser beam oscillation in circular and vertical eight shape. All three overlap welding strategies are presented with exemplary parameters in Figure 3.

The linear stitched weld is characterized by single lines of l = 3 mm length. These single lines were arranged at a horizontal distance of 1 mm, welded in alternating y-direction. In order to symmetrically distribute the energy input, the first seam is placed at x = 16 mm in positive y-direction, followed by two seams at x = 15 mm and 17 mm in negative y-direction. This stitching was then repeated 15 more times in alternating y-direction. The laser beam trajectories of the circular and vertical eight oscillation can be characterized by Equations (1) and (2) respectively. The trajectory of the laser beam using circular motion in superposition to a global linear feed rate is described by Equation (1)

(1) $$\left(\begin{array}{c} x\left(t\right)\\ y\left(t\right) \end{array}\right)=\left(\begin{array}{c} v_{f}\cdot t+a\cdot \cos\left(2\pi \cdot f\cdot t\right)\\ -a\cdot \sin\left(2\pi \cdot f\cdot t\right) \end{array}\right)$$

where $v_{f}$ is the linear feed rate, f the oscillation frequency, *a* the oscillation amplitude, and t the time [54]. The oscillation amplitude corresponds to the radius of the circular motion, visible in case (b) of Figure 3 in y-direction. The absolute velocity of the laser beam *v_p_(t)*, which corresponds to the feed rate of the linear stitched seam, is described by Equation (2).

(2) $$v_{p}=\sqrt{{\left(v_{f}-2\pi \cdot f\cdot a\cdot \sin\left(2\pi \cdot f\cdot t\right)\right)}^{2}+{\left(-2\pi \cdot f\cdot a\cdot \cos\left(2\pi \cdot f\cdot t\right)\right)}^{2}}$$

It becomes apparent that this value is not constant, in fact it varies along the weld trajectory, with the absolute highest speed in the direction of the linear feed rate and the lowest in the inverse direction [23]. The calculation of the magnitude of the path velocity therefore results in a sinusoidal course that oscillates around a mean value $\bar{v_{p}}$. The simplified relationship to the energy per unit length *E_L_*, which is directly proportional to the path velocity, can consequently be described mathematically by Equation (3)

(3) $$E_{L}=\frac{P_{L}}{\bar{v_{p}}}$$

where *P_L_* indicates the laser power on the material surface. When considering the time-dependent values $v_{p}\left(t\right)$ and $E_{p}\left(t\right)$, a detrimental effect caused by the asymmetry of the laser beam trajectory becomes apparent. Due to the local difference in $E_{p}\left(t\right)$ between the two sides in y-direction (y = ±0.75 mm), the weld penetration depth may be increased in areas with higher $E_{p}\left(t\right)$ compared to areas with lower $E_{p}\left(t\right)$ (inclination transverse to the global feed direction) [22]. Therefore, the vertical eight oscillation strategy is of interest because it can be used to achieve a symmetrical energy deposition relative to the global feed direction (*x*-axis). The trajectory of the laser beam using a vertical eight oscillation movement is described by Equation (4)

(4) $$\left(\begin{array}{c} x\left(t\right)\\ y\left(t\right) \end{array}\right)=\left(\begin{array}{c} v_{f}\cdot t+a\cdot \sin\left(2\pi \cdot f\cdot t\right)\\ a\cdot \sin\left(\pi \cdot f\cdot t\right) \end{array}\right)$$

For the comparison of the oscillation strategies, the parameters introduced above were used to calculate the degree of overlap U. This parameter is defined as the quotient of the distance between the local maximum of a first oscillation period *x_Max_*(*p*1) and the local minimum of the following second period *x_Min_*(*p*2) and the distance between the maximum and the minimum of the first period in global feed direction [23]. The relationship is described mathematically by Equation (5)

(5) $$U=\frac{x_{Max}\left(p1\right)-x_{Max}\left(p2\right)}{x_{Max}\left(p1\right)-x_{Min}\left(p1\right)}\cdot 100\%$$

wherein the spatial expansion of the laser beam is not considered. The analytic solution for the circular beam oscillation is shown in Appendix A. As discussed above, for the energy per unit length $E_{L}\left(t\right)$, a lower degree of overlap leads to a stronger asymmetry with regard to the global feed direction, since the position of the local maxima is no longer close to the y = 0 line.

The amplitude of the welding strategies with spatial beam oscillation was set to a fixed value of a = 0.75 mm for the investigations. Note that this value is smaller than the seam width of the linear stitched weld in order to compensate for lower values of the total length of the laser beam trajectory. The beam oscillation was applied with a maximum frequency of 250 Hz to ensure that the dynamic performance limit of the galvanometer scanner is not exceeded. This value was determined as threshold value from preliminary laser marking investigations on steel plates to evaluate the performance of the welding optics. The total weld seam length in feed direction (x) was set to 32 mm.

### 3.3. Experimental Design and Procedure

A total amount of 350 Cu and Al plate pairs was available for the investigation of the seam properties of overlap welding strategies from copper to aluminum in this work. In preliminary tests, process parameters were developed for all three welding strategies which ensure good connection quality, assessed first by visual inspection of the generated weld seams and followed by metallographic analysis. The weld seam quality was evaluated according to DIN EN 1011-6 [55]. If no connection in the overlapping area or if an excessive energy input was achieved (weld seam completely penetrates the Al-sample), the process parameters laser power and feed rate (or frequency respectively for oscillation strategies) were subsequently carefully adjusted. Two center point settings for each welding strategy were developed, listed in Table 4.

In the main test series, the size of the process windows for the determined process parameters #1–6 was then evaluated by systematic adjustment of the individual process parameters in small increments. For this purpose, metallographic and mechanical as well as electrical seam characteristics of the welded specimens were determined in the following in order to compare the joining strategies against each other. The analysis methods used are described in detail in the following section. The parameter sets were also tested for reproducibility, with each parameter set welded at least three times.

### 3.4. Electrical, Metallurgical and Mechanical Characterization

**Figure 4:**
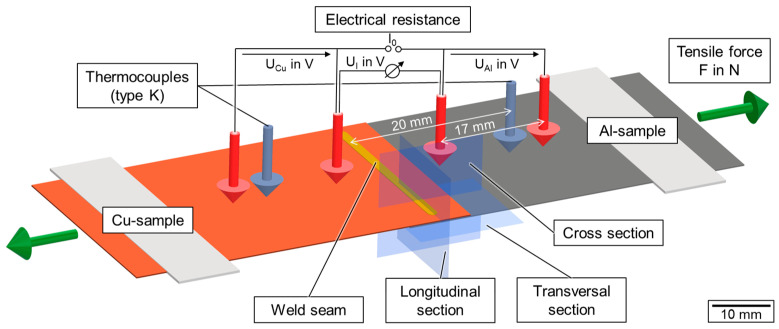
Schematic representation of the analyses carried out on the welded samples within the scope of the investigations.

Figure 4 shows a schematic representation of the analyses carried out on the welded samples within the scope of the investigations.

As outlined in Section 3.1, temperature measurement in the seam adjacent zone were conducted using two thermocouples (grey arrows) to record the maximum temperatures in the base metal during welding. The seam topography was recorded by an optical 3D profilometer (Keyence VR-6000, Keyence Corporation, Osaka, Japan) capturing the entire weld seam surface with a resolution of 0.1 μm in height direction before further destructive testing.

The electrical resistance measurement was executed by a combined measurement of the voltage and current as suggested by Ohm’s law [56]. Therefore, a 4-wire-measurement setup was built to introduce a defined current through the welded samples and to measure the voltage in equidistant distances of 17 mm in the base materials and the connection area (red arrows). A Sefelec MGR10 (Eaton Sefelec GmbH, Achern, Germany) resistance measurement unit working in the measurement range of 3 mΩ at 100 nΩ resolution with a measurement current of *I*_0_ = 1.0 A was used. The measurement accuracy is specified as ±0.1%. From the voltage drop U_I_ through the welded area, the connection resistance R_I_ of the welded area was calculated by Equation (6)

(6) $$R_{I}=\frac{I_{0}}{U_{I}}$$

For a material-independent evaluation of the electrical properties of the interconnection, the contact quality index (CQI) was calculated, which relates the proportion of the joint’s resistance to its base materials and dimensions [57]. The CQI is described mathematically by Equation (7)

(7) $$CQI=\frac{2\;\cdot R_{I}}{R_{Cu}+R_{Al}}$$

The electrical resistance values were determined from averaging ten discrete measurements. This method was applied three times per sample to cover the beginning, the middle area, and the end of the weld seam. These values were once more averaged to a median value including standard deviation. Since the resistance of the interconnection area is substantially affected by the number of intermetallic phases in the weld seam (see Section 2.2), these should be reduced to a minimum in order to achieve the lowest possible electrical resistance.

Metallographic examination was performed by means of longitudinal sections and cross sections of the weld seams as well as transversal sections of the interface area for selected samples. The view definition is illustrated by the example planes in Figure 4. Microstructural images were taken with an optical microscope (Olympus BX53M, Olympus K.K., Tokyo, Japan), measuring the interface weld width and penetration depth in the Al-sample. Therefore, the ground and polished sections were etched with a 5% Na-OH solution for three minutes. Intermetallic phase analysis was performed using a scanning electron microscope (SEM, Merlin Gemini II, Carl Zeiss AG, Oberkochen, Germany). To investigate the distribution of copper and aluminum in the seam after welding, energy-dispersive X-ray spectroscopy (EDS) mapping was applied. In addition, hardness measurements were conducted on the cross-sections with 1 N imprints (HV 0.1) using a hardness testing machine (KB 30 S, KB Prueftechnik). The mechanical testing of the seam performance was carried out according to DIN EN ISO 6892 [58]. The tensile force was applied perpendicular to the weld seam using a Quasar 100 tensile testing machine (Galdabini SA, Cardano Al Campo, VA, Italy). Two metal gaskets were placed underneath at both ends in the clamping jaws to ensure the coaxial application of the tensile force into the specimen to be tested. The preload was set to 10 N and the test rate was 10 mm/min. The fractured samples were analyzed by microscope and divided into seam failure (fracture in the weld seam) and a fracture in the base metal (aluminum plate).

## 4. Results and Discussion

### 4.1. Evaluation of Weld Seam Cross-Sections—Influence of Process Parameters

Since the series of experiments were designed to study the seam properties and process stability for different overlap welding strategies using green laser radiation for dissimilar copper to aluminum joints, investigations were first carried out to determine the suitable parameter sets for all three welding strategies.

#### 4.1.1. Linear Stitched Weld

**Figure 5:**
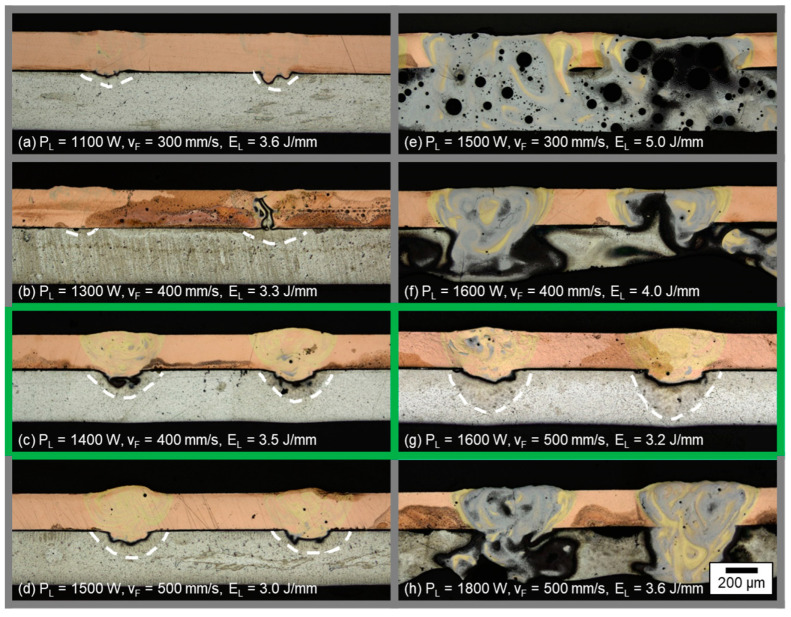
Longitudinal sections of linear stitched welds for different laser powers and feed rates.

In Figure 5 images of micrographs of longitudinal sections from joints produced with the linear stitched welding strategy at varied laser powers P_L_ and different feed rates v_F_ are shown. The resulting line energy is also depicted in the image captions.

In general, two types of weld seam appearance were observed in the micrographs. The first type includes either no or only minimal energy input detected in the aluminum (see white dotted lines for (a,b) as well as the excessive mixing in the micrograph due to excessive energy input (e,f,h)). Instead, the second variant reveals sufficient weld depth into the aluminum and limited intermixing (indicated by the dark grayish and golden colored areas in the longitudinal sections) in the copper aluminum interface, which is outlined by green lines in Figure 5c,g.

As expected, the weld depth into the aluminum sheets increased with increasing laser power at constant feed rates. Thereby, the fused area of aluminum and the mixing of both metals also increased. Pores were observed in the weld seams, predominantly located in the aluminum sheets as well as in the combined melt pool, as evident from sample (e). Cracks were found in welds with excessive energy input (E_L_ ≥ 3.6 J/mm) in the area of the resolidified molten material (see (e,f,h)). For lower line energies, no cracks were observed. Line energies in the range of 3.2 ≤ E_L_ ≤ 3.5 J/mm showed a sufficient interconnection area of copper and aluminum; however a minimum intensity must be applied to achieve a suitable weld seam depth, as in case (a,b) the laser power was too low to fully weld the aluminum sample (weld seams were not mechanically stable).

**Figure 6:**
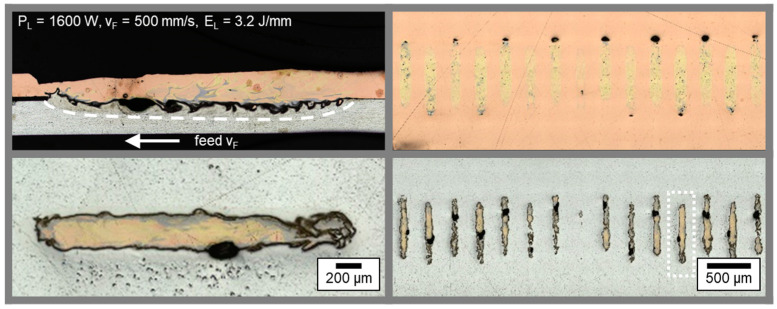
Micrographs (cross section and transversal section) of Cu-Al joint welded with P_L_ = 1600 W and v_F_ = 500 mm/s.

The interface area between copper top sheet and aluminum bottom sheet for the parameter set shown in (g) was furthermore analyzed with regard to seam morphology by preparing a cross and transversal section of the weld seam. The resulting micrographs are presented in Figure 6.

From the cross section at the top left, the evolution of the weld seam in feed direction can be observed. First of all, an increasing weld depth in the aluminum sheet is present due to heat accumulation in the interaction zone for the limited material thickness of the sample sheets. The intermixing of Cu and Al then increases until the end of the single weld seam, which can be identified through the sagged seam surface of the copper sheet. A constant weld depth was observed over the most part of the seam, caused by the high feed rate applied for the linear stitched weld. The dark area between the two joining partners indicates a layer of Al-rich intermetallics that have been reacting to the etchant.

The homogeneity of the weld seams produced with the linear stitched strategy especially becomes visible from the transversal-sections in copper and aluminum (Figure 6 right and detail bottom left). In the copper sheet, a constant weld seam width and length is observable. The high absorptivity of the green laser radiation in copper enables this reproducible weld seam quality for small aspect ratios [59]. End craters were found, which may be caused by the instantaneous shutdown of the laser power at the end of the weld seam. The solidifying melt shrinks (solidification shrinkage of copper is higher than for aluminum, [60]) and since no further liquid metal is available due to the completed welding process, an end crater can form at the weld seam end. The weld seam width in aluminum is found to be lower compared to the copper layer due to the high amount of heat conducted in the copper sample and the V-shape of the weld seam (see Figure 5). Apart from the first seam placed at x = 16 mm (middle area of the bottom right micrograph), a regular appearance of the linear stitched seams can be confirmed. A slight increase in seam width is observable in feed direction, which can be attributed to the reason stated above.

The unmodulated, linear welding of the dissimilar metals copper itself and aluminum is a highly challenging process and prone to defect formation and fluctuations in penetration depth [61]. It can be concluded that, compared to the investigations in [62], a constant seam quality in rectilinear welding was achieved by applying a special linear stitched welding strategy to symmetrically distribute the energy input in the material.

#### 4.1.2. Circular Beam Oscillation

**Figure 7:**
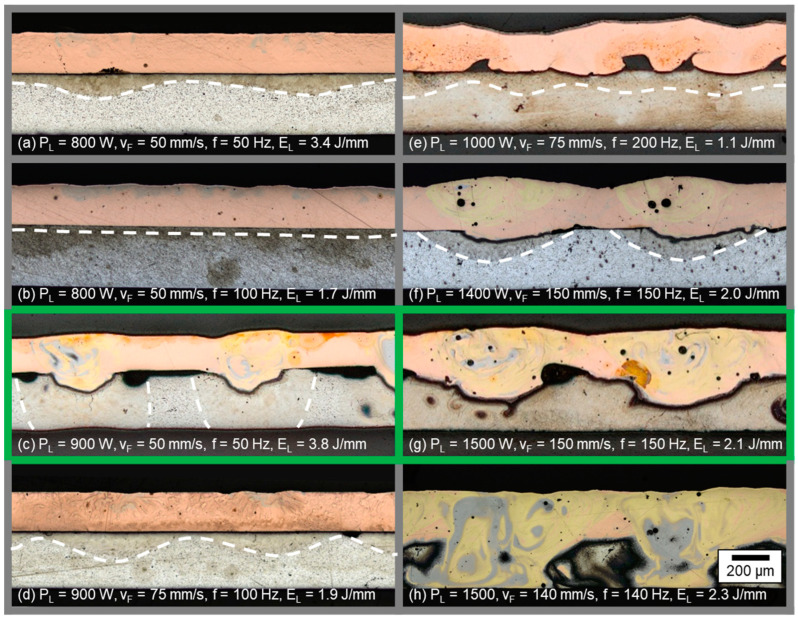
Longitudinal sections of welds produced with circular beam oscillation for different laser powers and feed parameters (v_F,_ f); welding direction from left to right.

In Figure 7 images of longitudinal sections from welds produced with circular beam oscillation at varied laser powers P_L_ and different feed parameters (v_F,_ f) are shown. The resulting line energies, calculated according to Equation (3) are added in the image captions.

As introduced in Section 4.1.1, two types of weld seam appearance can again be distinguished: Weld seams with insufficient energy input into the aluminum, where a wavy, darkened area can be detected in the aluminum, indicated by the white dashed lines in case (a), (b), (d), (e), and (f). The formation of these waves can be explained by the oscillating circular motion of the laser beam. As a result, the energy input and thus the penetration depth varies locally. In the case of excessive energy input (micrograph (h)), an intermixing over the entire height of both welding partners is visible. Nevertheless, areas with identifiable aluminum base material remain between the oscillating motion of the laser beam, observable in the middle and left area of micrograph (h). This fact is attributed to the low degree of overlap of ≈20% for this parameter set. The seam porosity appears to increase with increasing laser power, as only the longitudinal sections with P_L_ > 1000 W and visible metallurgical mixing of copper and aluminum are found to have pores in the weld seam.

Sufficient weld depth and limited mixing in the interface was achieved for parameter sets (c) and (g), which are outlined in green in Figure 7. For the lower feed rate and laser power (case c), the joining area is limited to a width in the range of the laser spot diameter. The heat-affected zone caused by the increased line energy (E_L_ = 3.8 J/mm) is clearly visible in the oval areas of the aluminum base metal. However, compared to the linear stitched weld strategy, a good metallographic joint quality was achieved with an absolute velocity ($\bar{v_{p}}$ = 236 mm/s) that was significantly lower compared to the parameter sets discussed in Section 4.1.1. The parameter set (g) using increased laser power and feed rate compared to (c) resulted in a more uniform mixing of the components. Due to the higher intensity on the copper sample and the accelerated beam movement (factor 3 compared to (c)), the continuity in the connection zone was increased at a reduced line energy.

In sum, the line energy range for proper welding parameters was found larger for the circular beam oscillation for a degree of overlap of U ≈ 20% compared to the linear stitched weld. For E_L_ = 2.1 J/mm, a controlled intermixing of copper and aluminum with distinct penetration depth was still achieved. This phenomenon is attributed to the spatial power modulation, significantly increasing the absolute point velocity of the laser spot but enabling a stable weld due to the special beam movement strategy. A further increase in the degree of overlap with the comparably large laser spot used in this study compared to a single mode beams, as investigated for example in [25], resulted in an excessive amount of Cu-Al-intermixing.

**Figure 8:**
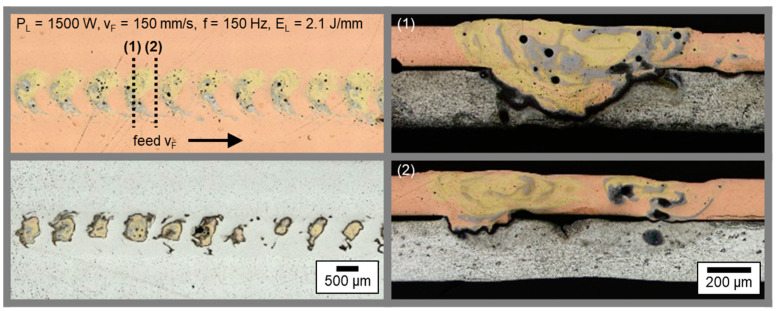
Micrographs (transversal sections (**left**) and cross sections from the planes of the dashed lines (1) and (2) of the transversal section top left (**right**)) of Cu-Al joint welded with circular beam oscillation at P_L_ = 1500 W, v_F_ = 150 mm/s, f = 150 Hz.

The results denote that, by means of an appropriate tuning of the process parameters, several different welding results can be achieved, demonstrating a good versatility of the process using green laser radiation. In addition, it becomes obvious that the feed rates used are suitable to avoid heat accumulation effects during welding, which occur when the heat flow is faster than the heat source and the material being joined tends to preheat gradually during the process [48]. No significant increase of the weld seam width and penetration depth was observed from the beginning to the end of the weld, as illustrated in Figure 8. Comparable to Figure 6, the left-hand side shows micrographs in sheet thickness direction in copper and aluminum for parameter (g).

The circular oscillating motion becomes particularly visible in the transversal sections where in the overlapping areas significant intermixing is observed in regularly appearing golden-colored areas (see Figure 8 top left). This phenomenon continues downwards into the aluminum sheet, as predominantly in these seam regions the local energy input is increased compared to the residual weld seam (Figure 8 bottom left). Two views in feed direction are shown in Figure 8 on the right side for different x-positions, revealing the locally varying seam width and penetration depth in detail. Due to increased energy input around y = 0 (see Figure 3), the element mixing is remarkable, and the weld seam nearly penetrates almost the complete aluminum sample in the case of cross Section 1. For cross Section 2, a reduced interface width, divided into two parts, and a lowered penetration depth become apparent. The more pronounced weld seam on the left side is related to the crossing point of the laser spot trajectory, which resulted in an increased energy input there. Overall, a good homogeneity and reproducibility of the presented process parameters with circular beam oscillation was found.

#### 4.1.3. Vertical Eight Beam Oscillation

**Figure 9:**
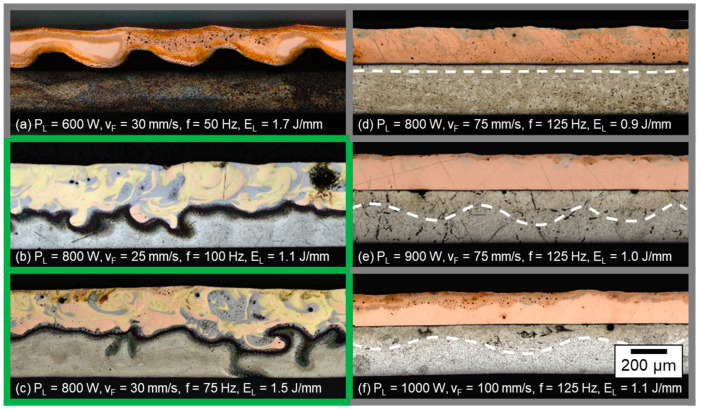
Longitudinal sections of welds produced with vertical eight circular beam oscillation for different laser powers and feed parameters (v_F,_ f); welding direction from left to right.

The investigated process parameters and corresponding longitudinal sections from welds produced with vertical eight beam oscillation at varied laser powers P_L_ and different feed parameters (v_F,_ f) are shown in Figure 9. As can be noted from the calculated line energies, lower values were found to be sufficient for this process strategy due to the high degree of overlap in the beam movement. For a good weld quality—outlined in green—a comparable value range of the line energy as for the linear stitched weld was found; however the E_L_-values of the latter had an offset of +2 J mm.

Similar to Section 4.1.2, weld seams with vertical eight oscillation also show a wave -like heat input into the aluminum that follows a regular repetition if the energy input was insufficient for metallurgical intermixing. As can be derived from the parameter sets (d) to (f), the amplitude and frequency of this wavy structure can be controlled by the corresponding feed parameters v_F_ and f. In the case of the weld seam in (a), the intensity was too low for a stable penetration of the keyhole into the aluminum, so the result did not show good mechanical properties. The copper layer is observed regularly bent upwards in the interface area, probably due to distortion.

A sufficient weld depth with component mixing in the interface area was found for parameter sets (b) and (c). Here, the lower feed rate and line energy setting case (b) showed higher regularity compared to the weld seam presented in Figure 9c. However, the higher degree of dark grayish and golden colored areas in the longitudinal sections indicates more intense melt flow dynamics and intermixing during welding for this oscillation strategy. In contrast to the circular beam oscillation, no pores or cracks were detected in the joints. It is assumed that this behavior is related to the low line energies and high degrees of overlap (U(b) = 83%, U(c) = 74%) used for these experimental investigations.

**Figure 10:**
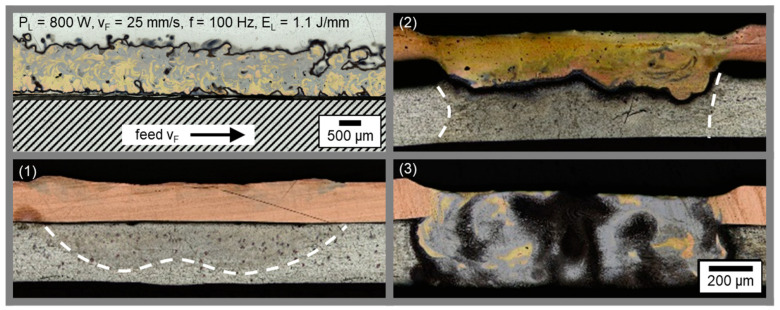
Micrographs (transversal section (top left) and cross sections at a distance of 2 mm (1), 16 mm (2), and 30 mm (3) from the starting point of the welding process) of Cu-Al joint welded with vertical eight beam oscillation at P_L_ = 800 W, v_F_ = 25 mm/s, f = 100 Hz.

Figure 10 shows the weld seam evolution in feed rate direction for parameter set b) from cross sections and a transversal section. A turbulent intermixing of copper and aluminum over the entire seam area in width direction can be identified top left. From the three cross sectional micrographs (1) to (3), the weld seam continuity of the vertical eight oscillation may be studied: these were prepared with a distance of 2 mm (1), 16 mm (2), and 30 mm (3) from the starting point of the welding process. At the beginning of the weld, hardly any mixing can be observed, which is attributed to the low laser power used for this parameter set (compare Section 4.1.1 and Section 4.1.2). The symmetry of the beam oscillation profile becomes noticeable in the heat affected zone in aluminum in the form of a W-shape. The intermixing of copper and aluminum increases as the laser welding process progresses and heat accumulation in the material becomes significant, see cross Section 2. A homogenous weld seam with slightly larger penetration depth to the sides can be observed. Toward the weld seam end, the energy input seems to accumulate and the intermixing over the entire height of both welding partners is visible. This phenomenon is attributed to the almost three times higher trajectory length compared to the circular oscillation strategy.

**Figure 11:**
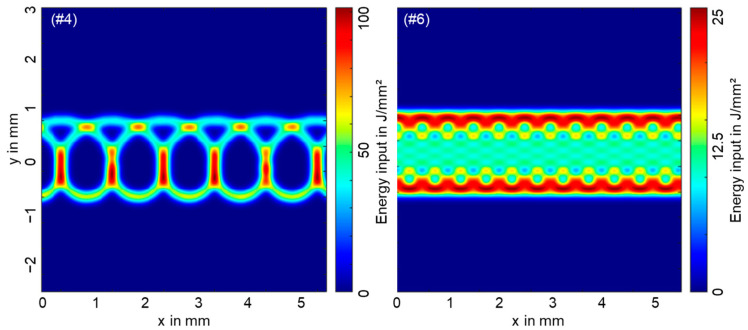
Distribution of the local energy input by spatial power modulation with a circular oscillating beam, P_L_ = 1500 W, v_F_ = 150 mm/s, f = 150 Hz (#4, **left**) and by a vertical eight oscillation strategy with P_L_ = 800 W, v_F_ = 25 mm/s, f = 100 Hz (#6, **right**).

The difference in distribution of the local energy input energy for the circular and vertical eight beam oscillation strategy is shown by analytical calculations in Figure 11. Here, the distribution of the locally deposited energy is calculated according to

(8) $$\int I\left(x\left(t\right),y\left(t\right)\right)dt$$

for a laser beam with incident power *P_L_* and the local intensity profile [63] of

(9) $$I\left(x,y\right)=\frac{8\;\cdot P_{L}}{\pi \cdot d_{F}{}^{2}}\cdot e^{-8\frac{\left|x{\left(t\right)}^{2}+y{\left(t\right)}^{2}\right|}{d_{F}{}^{2}}}$$

moving over the copper sample according to the given oscillation patterns.

It becomes clear that the two oscillation patterns produce significantly different distributions of the incident energy. Distinct peak areas of the deposited energy are present at the overlapping points along the circular beam oscillation (Figure 11 left). Here, the local energy introduced into the welding process (coupled into the vapor capillary) significantly exceeds the local energy deposited anywhere along the trajectory in vertical eight beam oscillation. The constant and fast movement of the beam along the vertical eight oscillation path results in a lower and a more evenly distributed energy deposition, as shown in Figure 11 right. However, the high degree of overlap in this case leads to a more areal energy input (see light blue and yellow areas).

Looking at the micrographs presented in Figure 7, Figure 8, Figure 9 and Figure 10, the weld seam appearance can be understood more conclusively. The high energy input in the middle overlapping area as well as at y = 0.75 mm can be correlated with the increased amount of intermixing and interconnection width in cross Sections 1 and 2 in Figure 8. In contrast, the lower region around y = −0.75 (Figure 11 left) and the right part of cross Section 2 (Figure 8) for the circular oscillation strategy show a lower energy input and thus a limited amount of metallic intermixing. The W-shape and the symmetrical applied energy input along the *x*-axis for the vertical eight oscillation are clearly reflected in the weld seam appearance in micrograph 2 in Figure 10.

The six weld seam results selected above (outlined in green) were used as reference parameters for the microstructural, mechanical, and electrical analysis and discussion below. The parameter sets are listed alongside the energy input (feedback from laser source) and beam trajectory characteristics in Table 5.

**Figure 12:**
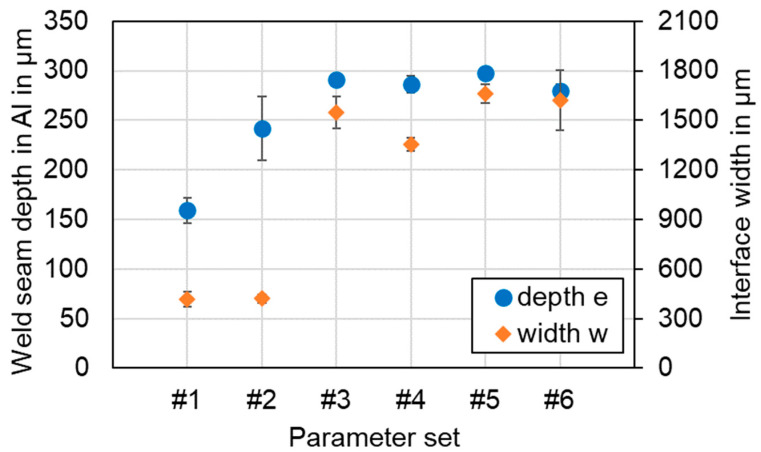
Weld seam depth in aluminum and interface width for selected parameter sets (*n* = 4).

Figure 12 shows the relationship between the parameter sets and the main geometrical characteristics of the weld seams. In general, the results confirm that the process parameters have a significant influence on the weld pool geometry and thus on the appearance of the copper aluminum interface.

A decisive influence of the line energy was found for the linear stitched welds, with a larger standard deviation for the higher laser power applied in #2, indicating increased process dynamics. The interface widths (in feed direction of the laser spot) of the two seams were observed comparatively small at about 25% of the mean value of parameter set #3–6. This fact is comprehensible due to the missing oscillation movement of the laser beam in these cases. Both sets of parameters of both oscillation strategies (circular and vertical eight) showed an almost complete penetration of the aluminum sheet in their mean values, whereas the interface connection width was between 1350 and 1660 µm. These values are in the range of the applied oscillation amplitude and indicate that a deep penetration welding has taken place, incorporating the entire metal in the oscillation path. Good reproducibility of the parameter sets can be determined by the low standard deviations. However, it must be taken into account that with the oscillating overlap welding strategies there is a significantly higher energy input into the joining partners, as the weld seams are continuous and altogether longer (see Table 5).

Comparing the presented results with those obtained by the authors in references [25,43], it becomes clear that deep penetration welding at a visible wavelength allows sound weld seams with a reasonable penetration depth control and process windows for linear and oscillating overlap welding strategies. In contrast, laser beam welding with near-infrared wavelengths requires the use of small beams (single mode beam quality) to achieve good welding results and a stable process window. Only small interface widths can be achieved, which can be extended by the application of spatial power modulation. The process parameters developed in this experimental study also favored very different mixing ratios between the base metals copper and aluminum. Mixing is minimal for low seam lengths and energy input in the case of the linear stitched welding strategy. This also corresponds to the short interaction times between laser and joining partners. In contrast, mixing is more evident in case of the oscillated welding strategies, as the larger melt pool promotes component intermixing.

### 4.2. Electron Dispersive Spectroscopy (EDS) Analysis Results—Influence of Welding Strategy

**Figure 13:**
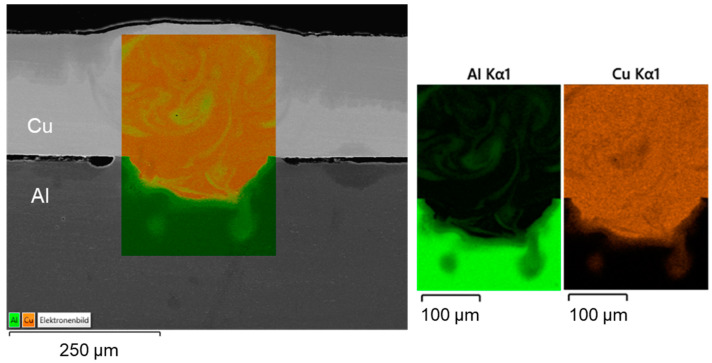
EDS mapping image for the joint cross-section of parameter set #2 (linear stitched weld, P_L_ = 1600 W, v_F_ = 500 mm/s) showing the elemental distribution of Cu and Al in the weld seam.

The high thermal gradient induced during laser beam welding favors the formation of various microstructures and intermetallic phases [64]. Three of the presented overlap joint samples were thus chosen for EDS analysis, where these samples were generated using linear stitched weld strategy (#2), circular beam oscillation (#4), and vertical eight beam oscillation (#6) respectively. In the surface scans, copper is colored orange and aluminum is colored green. Figure 13 shows the EDS analysis results (surface scan) for a linear stitched weld fabricated using parameter set (#2).

**Figure 14:**
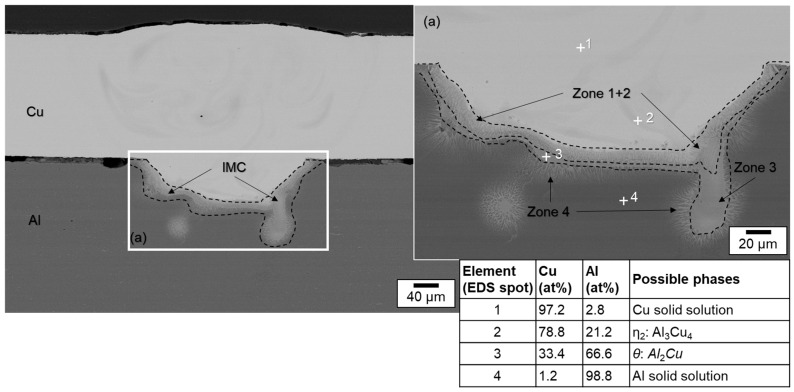
SEM image and EDS scanning spots in the weld seam of the sample produced with parameter set #2; white insert (a) indicates the area of the displayed detail image (a); dashed line indicates intermetallic phases (**top**); EDS phase composition at selected scanning spots (**right bottom**).

Slight mixing is present in the weld seam, especially upstreaming aluminum in the copper melt can be noticed from the element distribution. It is particularly evident that in the case of partial penetration, the limited amount of molten aluminum is distributed quite uniformly in the copper melt due to the significant difference in density between the two metals. Intermetallic phases were predominantly formed in a small area at the interface of copper and aluminum. These were detected in EDS scanning spots, see the EDS phase compositions at selected scanning spots in Figure 14. Observing the boundary region of the melt pool, it can be seen that spot 1 possibly consists of Cu solid solution, while spot 4 seems to have Cu solid solution [65]. However, spot 2 in the interface region of the weld seem to have Al_3_Cu_4_ phase and spot 3 Al_2_Cu phase, which may have an adverse effect on the joint strength [66]. The presence of numerous intermetallic phases in the interconnection area increases the risk of solidification cracks [65]. However, no obvious pores and cracks were detected in the fusion zone.

A subdivision of the intermetallic layer into zones can be made according to the investigations presented in [67]. Thereby, the formation of four different zones can be distinguished: in zone 1, the granular reticular structure is formed from the γ_1_-Al_4_Cu_9_ phase which is often the first to be formed during the reaction. Zone 2 is a mixture of eutectic and hypoeutectic structures. It is characterized by a needle-like structure. The measurements yielded the η_2_-AlCu phase, which contains about 50 at. % aluminum and copper. In zone 3, a eutectic structure of α-Al and mainly θ-Al_2_Cu is present. Zone 4 is characterized by a dendritic structure that forms near the aluminum base metal. The growth of the solidification front and the segregation of the alloying elements create the characteristic dendritic arms [67].

**Figure 15:**
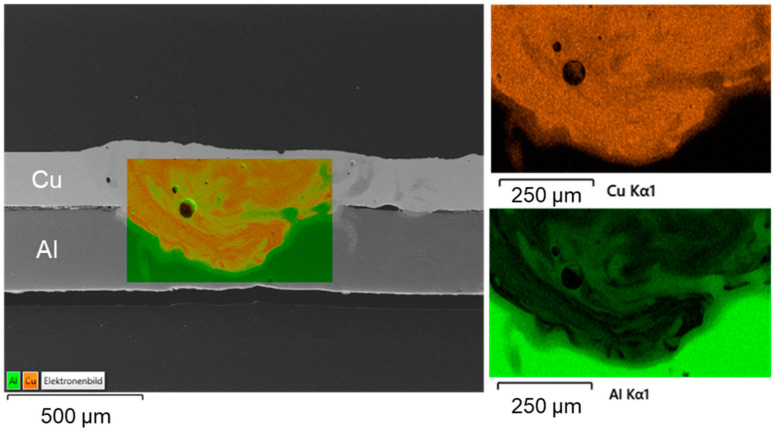
EDS mapping image for the joint cross-section of parameter set #4 (circular beam oscillation, P_L_ = 1500 W, v_F_ = 150 mm/s, f = 150 Hz) showing the elemental distribution of Cu and Al in the weld seam.

**Figure 16:**
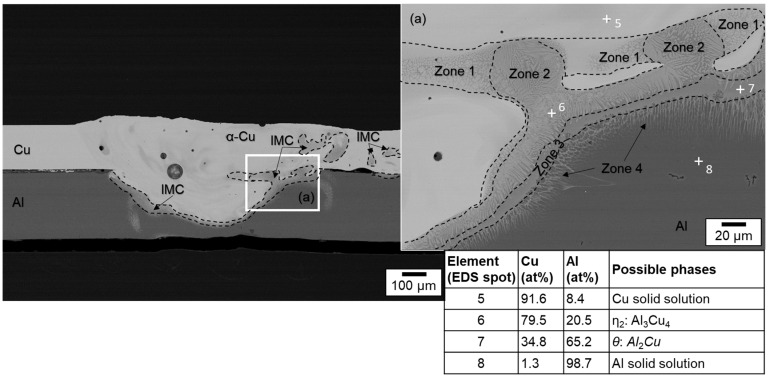
SEM image and EDS scanning spots in the weld seam of the sample produced with parameter set #4; white insert (a) indicates the area of the displayed detail image (a); dashed line indicates intermetallic phases (**top**); EDS phase composition at selected scanning spots (**right bottom**).

The EDS mapping results for a weld generated with circular beam oscillation using parameter set (#4) in Figure 15 show a non-uniform distribution of the copper and aluminum contents in the weld seam. This phenomenon has the potential to reduce the strength of the joint (see Section 4.5). The observed porosity could be attributed to the high oxygen affinity of the molten aluminum [68]. The spot measurements shown in Figure 16 in combination with SEM images of the weld seam revealed intermetallic phases of distinct types that were widely distributed across the weld seam. This distribution can cause high hardness values, which are examined in Section 4.4 in conjunction with Table 1. From Figure 16, it is obvious that the interface zone along the direction from pure copper to aluminum weld metal zone is composed of the copper solid solution in the position of point 5, Al_3_Cu_4_ in the position of point 6, Al_2_Cu in the position of point 7, and aluminum solid solution in the position of point 8 [65]. Due to the increased energy input (increased E_L_) compared to parameter set #2, intermetallic compounds were also detected near the copper top surface (especially visible in the right part of the seam, which belongs to the interior of the circular beam movement).

**Figure 17:**
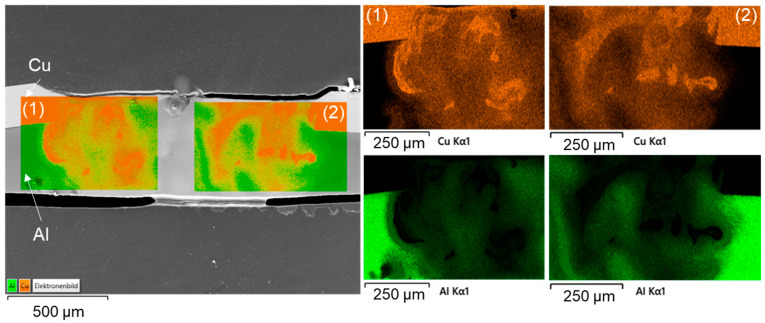
EDS mapping images (1) and (2) for the joint cross-section of parameter set #6 (vertical eight beam oscillation, P_L_ = 800 W, v_F_ = 25 mm/s, f = 100 Hz) showing the elemental distribution of Cu and Al in the weld seam.

**Figure 18:**
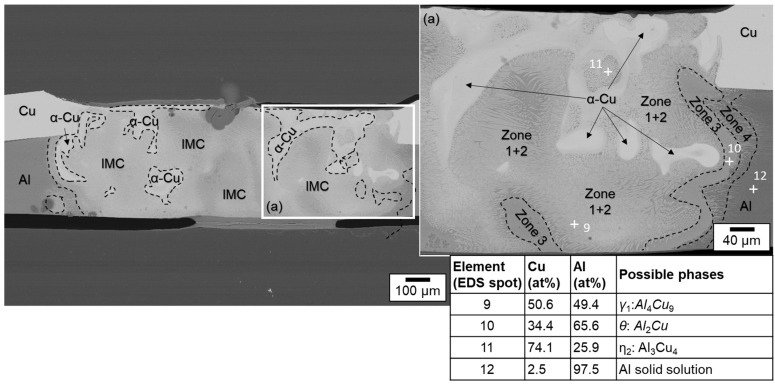
SEM image and EDS scanning spots in the weld seam of the sample produced with parameter set #6; white insert (a) indicates the area of the displayed detail image (a); dashed line indicates intermetallic phases (**top**); EDS phase composition at selected scanning spots (**right bottom**).

Similarly, the SEM morphologies and EDS results for the copper aluminum joint produced with the vertical eight beam oscillation and parameter set #6 are shown in Figure 17 and Figure 18. Compared to the linear stitched weld and the circular beam oscillation presented above, an excessive intermixing of the joining partners, as already discussed in Section 4.1.3, becomes visible. Copper is detected in aluminum down to the ground of the weld seam and, conversely, aluminum can be found in copper up to the weld seam top surface. Consequently, large amounts of intermetallic phases are observed in the weld seam, indicated by the outlined areas in Figure 18. In addition, it becomes apparent from the phase composition presented in Figure 18 that EDS spot 9 is likely to consist of Al_4_Cu_9_. The interface area is furthermore composed of Al_2_Cu in the position of point 10, Al_3_Cu_4_ in the position of point 11, and aluminum solid solution in the position of point 12 [65].

From the microstructural analysis of the fusion zones, it can be concluded that the absence of a beam oscillation significantly reduces the formation of intermetallic phases. The short interaction time at high intensity and feed rate limits the formed layer width for parameter set #2 to a thickness of ≈40 µm. In comparison, increased copper aluminum intermixing and subsequent formation of intermetallic phases were observed when using the circular beam oscillation strategy, which is in good accordance to the results presented in [56]. The largest amount of intermetallic phases was detected for the vertical eight oscillation strategy (parameter set #6). 

It should be noted that no significant amount of nickel was detected in the interface areas and fusion zones (<0.5 at. %). This fact can be attributed to the low plating thickness used in the investigations. Nevertheless, the incoupling behavior of the laser beam may be positively influenced due to the increased absorptivity of nickel compared to copper for λ = 515 nm [69]. Whether the idea of distributing the intermetallic compounds uniformly in the weld to achieve a ductile behavior of the seam was successfully implemented was investigated by tensile tests and contact resistance measurements. The analysis of weld seam performance under variation of welding strategy and processing parameters is presented in the following.

### 4.3. Seam Surface Roughness Analysis

**Figure 19:**
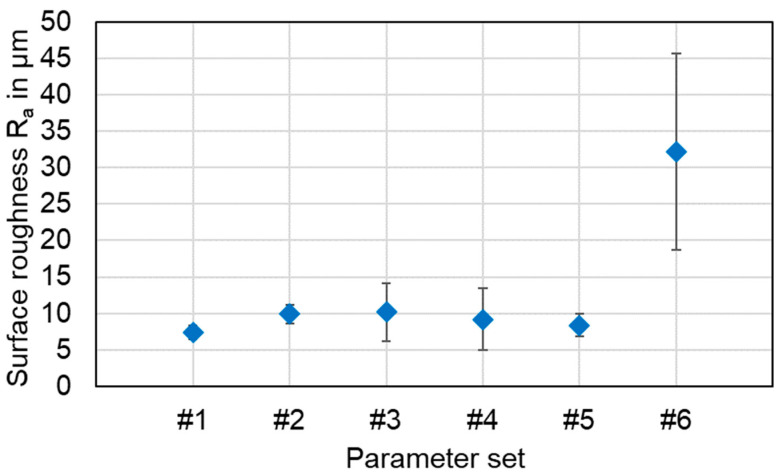
Seam surface roughness evaluated over the entire seam area on copper for parameter sets #1–#6 (see Table 5, *n* = 6).

The surface appearance of the weld seam in laser beam welding is a meaningful criterion, as it usually gives a good first impression of the weld quality through visual inspection. Therefore, the selected parameter sets were examined in terms of seam surface roughness to obtain a quantitative, comparable metric. An arithmetic mean of the surface roughness was calculated by averaging the Ra values from the entire weld seam of each specimen. The results are shown in Figure 19.

It is noticeable in this case that the surface roughness of sample #6 is significantly higher compared to all other sets of parameters examined. The highest value of 32.2 μm is reached for the vertical eight beam oscillation with P_L_ = 800 W, v_F_ = 25 mm/s, f = 100 Hz. These samples also had the highest standard deviation of 13.5 μm. This behavior is attributed to the high energy input associated with this parameter set, which leads to increased elemental intermixing and high dynamics in the interaction zone. In addition, this parameter setting shows the highest degree of overlap applied in the studies. The surface roughness for the linear stitched weld #1 was found to be the lowest, while the other parameter sets were observed with R_a_-values of about 10 µm. The increased dynamics when applying beam oscillation strategies are also reflected in the results of this analysis by an increased standard deviation.

In summary, the surface roughness was observed for five of six parameter sets with Ra values R_a_ ≤ 10.5 µm. With regard to the investigations presented in [70], comparable roughness values could be achieved for the surface quality, although a significantly larger spot diameter was used in the experimental studies presented in this work. By the application of the stitched welding strategy #1, an average roughness of R_a_ = 7.4 µm can be achieved, which may help to minimize rework efforts and reduce the required installation space for battery tab connections in potential applications.

### 4.4. Vickers Microhardness Analysis of the Weld Seams

The mechanical properties of the welded joints were evaluated through microhardness analysis and tensile tests at room temperature. This section presents the hardness measurements of the tree parameter sets #2, #4, and #6 with different welding strategies performed on cross sections of the weld seam (longitudinal section for the linear stitched weld respectively). A force of 1 N (HV 0.1) was applied for the imprints, while the distance between the imprints was set to 0.2 mm. Thus, for the oscillating welding strategies, eight imprints could be placed in the joint area. The base material on both sides was added to study softening effects after welding. For the linear stitched weld, microhardness values were obtained at five locations in and adjacent to the joint area. Two measurement routes were performed with a distance of 0.1 mm from the interface in copper and aluminum, respectively. Table 6 shows the microhardness values of unwelded copper and aluminum specimens as a reference.

**Figure 20:**
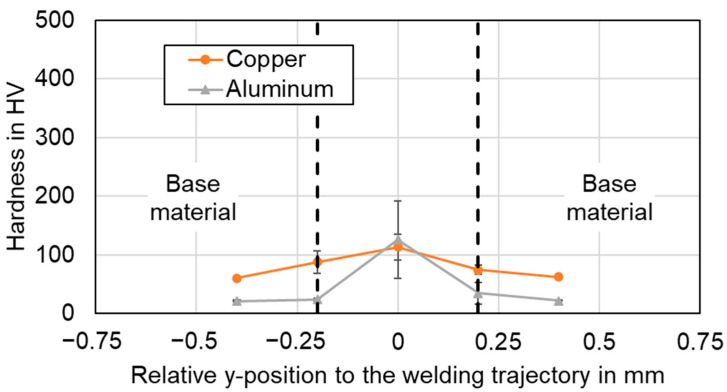
Microhardness profiles of the linear stitched seam fabricated with parameter set #2 (*n* = 5).

In Figure 20, the microhardness profiles of the linear stitched joint welded with parameter set #2 in copper and aluminum are displayed.

**Figure 21:**
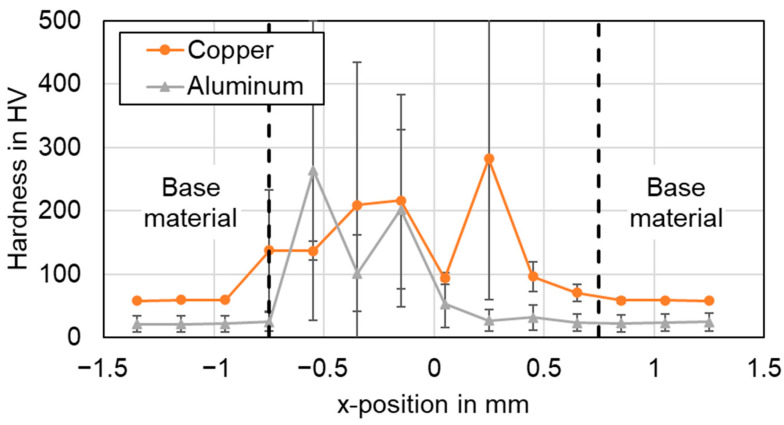
Microhardness profiles of the weld seam produced with circular beam oscillation using parameter set #4 (*n* = 4).

**Figure 22:**
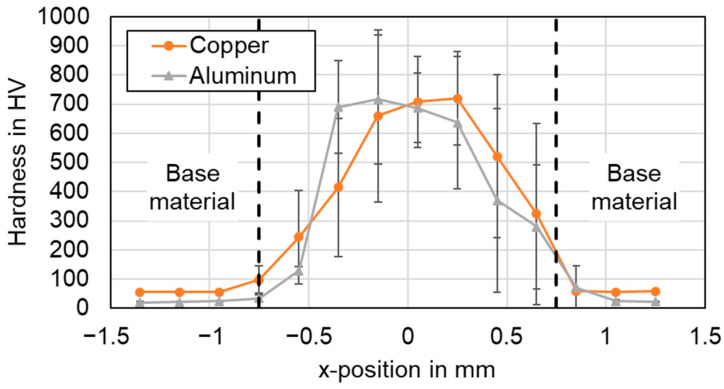
Microhardness profiles of the weld seam produced with vertical eight beam oscillation using parameter set #6 (*n* = 4).

Figure 21 and Figure 22 show the results for the circular beam oscillation using parameter set #4 and the vertical eight beam oscillation applying parameter set #6 respectively. Note that the hardness scales are different due to the maximum values observed.

In general, it is found that the hardness in the weld seams is significantly higher than the hardness in the heat-affected base material of both copper and aluminum. No significant softening of the base metal was observed in any of the examined weld seams. The actual hardness profile in the joint area was found to be significantly dependent on the choice of process parameters and welding strategy.

The highest hardness values for the linear stitched weld were determined in the center of the weld with almost 140 HV in copper and aluminum. This indicates controlled elemental mixing in the interaction zone, as increased microhardness values were observed in the weld seam, particularly significant for aluminum. The hardness profiles when applying temporal power modulation reveal a different behavior. The hardness curves of the circular beam oscillation show an average value of 155 HV for copper and 91 HV for aluminum in the seam cross section. The increased copper aluminum intermixing compared to the linear stitched weld can also be noted from the significantly increased standard deviation in Figure 21. A maximum microhardness of 300 HV for four averaged weld seams was observed, while peak values of up to 500 HV indicate the presence of copper-rich intermetallic phases in the joint. The vertical eighth oscillation strategy is characterized by the highest microhardness values measured in this study. Average values of 460 HV for copper and 440 HV for aluminum were determined for the seam cross sections. The maximum microhardness in this case was 720 HV in copper, with outliers near 1000 HV (967 HV for copper, x = −0.15). The plot of the hardness values for parameter set #6 denotes symmetrically distributed components at the position of copper and aluminum. This in turn points to large amounts of intermetallic phases in the weld seam. The observed behavior may have a negative effect on the current flow in the weld seam, which is discussed in Section 4.7.

In comparison, the average hardness across the weld seam area was the lowest for the linear stitched weld compared to the oscillating welding strategies. It was found that a high degree of overlap, as in the vertical eight beam oscillation, significantly increases the energy input and thus can strongly stimulate the formation of intermetallic phases, which are significantly harder than the base material (compare Table 1). The presence of intermetallic phases can be well correlated with the EDS measurements, as shown in Section 4.2, which also confirm their presence in the weld seams with varying amounts.

### 4.5. Tensile-Shear Strength Testing

**Figure 23:**
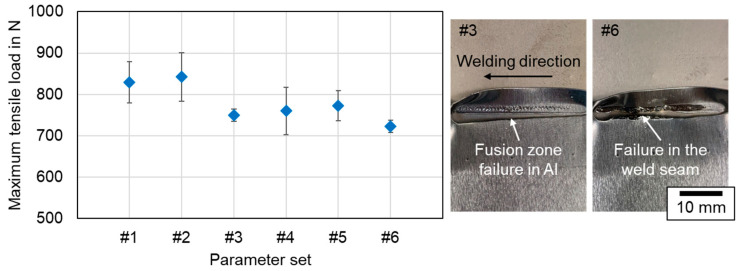
Tensile-shear strength result comparison for parameter sets #1–#6 (see Table 5).

The tensile strength of the joints was tested in a standard tensile shear test machine as described in Section 3.4. Thereby, the parameter sets #1–#6 with different overlap welding strategies and process parameters were investigated. In general, beam oscillation increases the component intermixture in the weld seam area and results in a larger connection width of copper and aluminum (compare Figure 12). The shear strength of the joints is reported in Figure 23 in terms of the maximum tensile load at failure. The exact resisting area depends on the failure location and the calculation of the absolute fusion area is tedious and depends on the seam geometry, which is why this representation was chosen here. Four samples were examined per parameter set in order to obtain statistically relevant results and to obtain information about the stability of the methods.

As can be seen in Figure 23, the processed samples with linear stitched welding strategy held the highest average loads of about 830 N (#1) to 840 N (#2), almost reaching the base metal strength of the aluminum sheet, which was measured to be 860 N (Cu: 2370 N). Their counterparts generated using beam oscillation withstood tensile forces of 700 N to 800 N on average. Thus, a reduction of about 100 N can be derived for all oscillating welding parameters compared to parameter sets #1 and #2. The standard deviation remained limited, with the highest value reaching 58 N for the circular beam oscillation in parameter set #4. The photographs in Figure 23 on the right show the failure behavior in the tensile test for a circular and a vertical eighth oscillation parameter, respectively.

All specimens, except from the vertical eight beam oscillation parameter #6, fractured in the aluminum base metal in the softened region within the heat-affected zone and not in the molten seam area. These results are in good agreement with the findings presented in [25]. In the case of parameter set#6, a failure in the weld seam in aluminum occurred, which is assumed to be related to the large number of intermetallic phases in the weld seam. The distribution of these phases with a combination of ductile and brittle intermetallic structures apparently was achieved with parameter set #5, as the average tensile strength of this parameter set was found to be the highest for the oscillating welds. This outcome confirms the studies in [49], where this type of oscillation is also found to be beneficial for the distributing of the intermetallic phases, since the fluid flow and the resulting fusion zone follow the trajectory of the laser beam. For the linear stitched weld, a higher maximum tensile load could be achieved in case of #2 with lower line energy. This behavior follows the explanations in [49], describing an increasing tendency of the shear strength with increasing laser power up to a maximum value for a linear weld, after which a further power increase activates more copper and aluminum and thus leads to a larger fusion zone with simultaneously decreasing shear force.

In summary, linear stitch welded specimens exhibit good mechanical stability and were able to withstand higher forces than specimens welded by use of beam oscillation when processed with the same experimental setup using a 340 µm laser spot with green laser radiation at keyhole aspect ratios of ~1.

### 4.6. Temperature Measurement in the Seam Adjacent Zone during Welding

**Figure 24:**
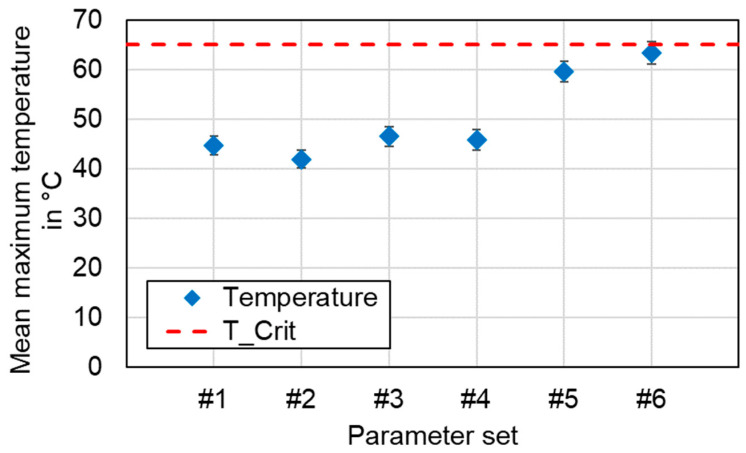
Mean temperatures occurring in the seam adjacent area during welding for parameter sets #1–#6 (see Table 5).

In Figure 24, the averaged maximum temperatures (*n* = 4) occurring in the seam adjacent zone during the welding process are shown. All measured temperatures in the seam adjacent zone were below the maximum permissible temperature of *T_Crit_* = 65 °C. At a distance of 20 mm between the symmetry plane of the weld seam and the measuring points, a maximum temperature of 63 °C was detected for parameter set #6 on the copper tab side. In general, the maximum temperature was recorded in the copper sheet, as can be seen, for instance, in the diagram in Section 4.10 which was attributed to the upper positioning and the high thermal conductivity of copper. Since the welding process takes only about one second of time, a rapid heating of the welded samples takes place, followed by different cooling curves for both metals. This high thermal gradient favors the formation of intermetallic phases in the weld seam area [64] but also leads to the fact that the seam adjacent zones stayed below *T_Crit_*. The evaluation of the influence of process parameters on the mean maximum temperature in the battery tab connections shows values between 40 °C and 50 °C for the linear stitched weld and the circular beam oscillation. In both cases, a reduced line energy has a decreasing effect on the value trend. The increased degree of overlap in parameter set #6 compared to #5 results in an increase in the maximum temperature in the seam adjacent zone due to an increased heat accumulation effect.

Based on the measurement results, it is recommended for the application of laser-based contacting of battery tab connections to ensure a minimum distance of 20 mm between the center of the weld trajectory and the critical component (e.g., the current collector edge).

### 4.7. Electrical Connection Resistance

**Figure 25:**
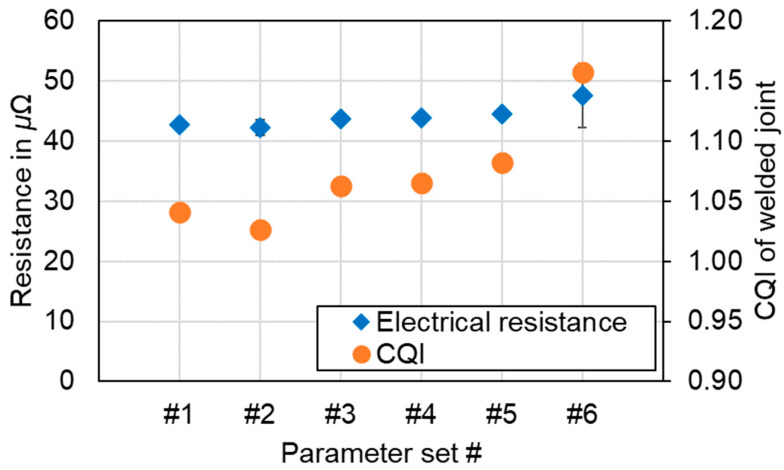
Electrical contact resistance and CQI of the specimen welded with parameter sets #1–#6 (see Table 5).

Figure 25 shows the results for the electrical connection resistance measurements of the welded joints. For this purpose, the average values of ten resistance measurements on four samples per parameter set (*n* = 4) were calculated. Each welded copper aluminum sample was evaluated at three distinct locations, at the beginning, in the middle, and at the end of the weld seam respectively. As no significant trend was observed along the path of the weld seam, only the averaged values and standard deviation are shown in Figure 25.

It should be noted that all measured resistances were between the two reference values of the base materials (R_Cu_ = 28 μΩ and R_Al_ = 55 μΩ). This behavior confirms the fact that all welding conditions studied promote the formation of solid joints that do not significantly affect the electrical performance of the final joint, either by the shape of the weld seam or by the increased formation of intermetallic phases.

As can be noted from Figure 25, the resistance values for the linear stitched welds were below 45 μΩ. In addition, a slightly improved resistance with reduced line energy can be detected between parameter sets #1 and #2. The lowest resistance was measured at 42.2 μΩ for a laser power of P_L_ = 1600 W at a feed rate of 500 mm/s (#2). For the circular beam oscillation strategy, the resistance of both parameter sets was in the range of 43 μΩ to 44 μΩ. The resistance of the first parameter set of the vertical eight beam oscillation (#5) was 44.5 μΩ, which is also within the range of the other two overlap welding strategies. The highest resistance was found for #6 while the CQI was 1.16. This significant increase in resistance can be explained by an increased amount of intermetallic phases in the weld seam, already discussed in Section 4.2. All other parameter sets examined could achieve values close to a CQI of 1, emulating the conductance of the base materials. Moreover, the difference between the resistance values of parameter sets #1–#6 is less than 13%. This result confirms that all processing conditions used within this work are suitable for achieving proper electrical connections between dissimilar battery tab connections. The obtained resistance values are comparable to those obtained by the authors in [43,71], proving that overlap welding of copper to aluminum using green laser radiation and a comparatively large laser beam can achieve joint properties similar to those obtained when near-infrared single-mode lasers are used.

**Figure 26:**
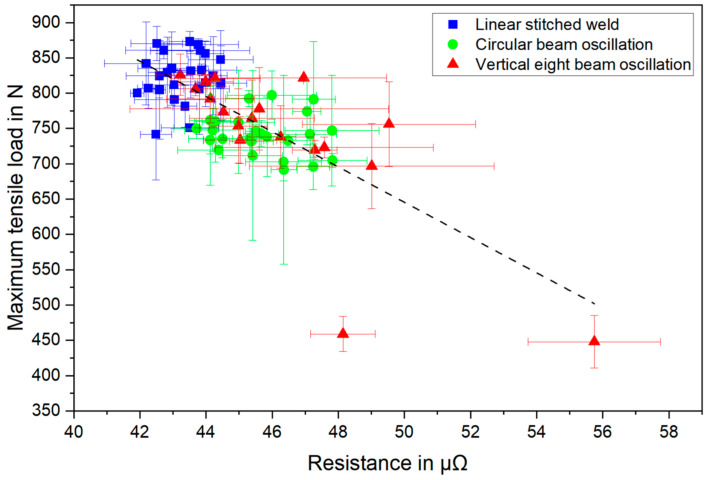
Correlation between tensile strength and electrical resistance for welds produced with linear stitched welding strategy (blue), circular beam oscillation (green), and vertical eight beam oscillation (red); error bars indicate the standard deviation; dashed line indicates the overall trend.

In addition, the tensile shear strength values from Section 4.2 and Section 4.8 were correlated with the measured electrical resistances of the weld seams. In Figure 26, both the maximum tensile loads together with the electrical resistances are plotted for the welds produced with and without spatial beam oscillation.

As discussed in Section 4.5, maximum loads of up to 880 N were observed, which coincide with the lowest electrical resistance of 42 μΩ. The mechanical and electrical quality were found to be correlated, thus the lowest electrical resistances were measured for high tensile loads and vice versa. From the accumulation of measurement points, it can be concluded that the linear stitched welding strategy has the best mechanical and electrical properties with comparatively small scattering. This is followed by the circular beam oscillation. The widest distribution was observed for the vertical eight beam oscillation. Therefore, process optimizations that address one of these quality parameters are expected to simultaneously optimize the other. In addition, the non-destructive measurement of the electrical resistance may allow the mechanical strength of the joint to be predicted (see overall trend for all parameter sets indicated by black dashed line). Therefore, the fast and reliable determination of the joint resistance is assumed to be beneficial for the high-scale manufacturing of battery tab connections. This topic is addressed by a clamping device with integrated sensor technology for direct data acquisition before, during, and after the welding process close to the interaction zone, see Section 4.10. In sum, presented results agree well with correlations reported for the thin sheet welding using a single-mode fiber laser in [72].

### 4.8. Process Window Evaluation—Statistical Analysis of Parameter Variations

**Figure 27:**
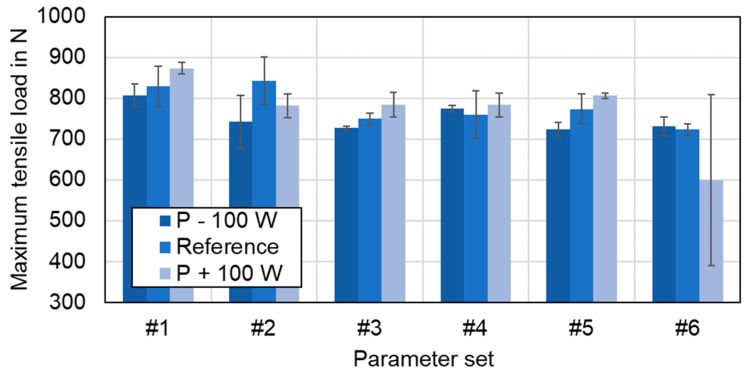
Comparison of tensile-shear strength results for parameter sets #1–#6 and variation of the laser power.

**Figure 28:**
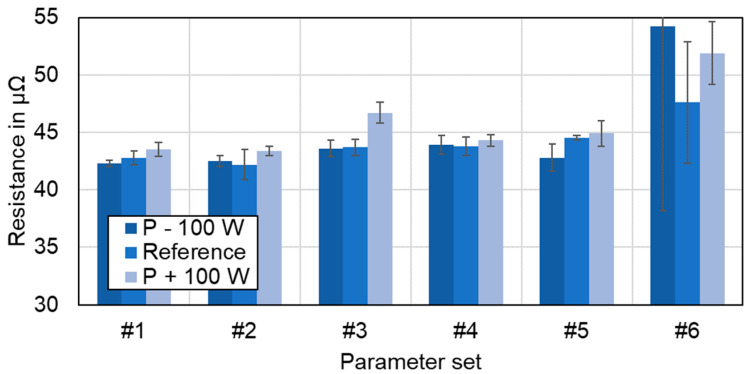
Comparison of electrical contact resistance measurements for parameter sets #1–#6 and variation of the laser power.

In order to investigate the process stability of the three different overlap welding strategies, investigations with laser power variation from reference parameters #1–#6 were conducted. For this purpose, offset values of 100 W higher and lower laser power compared to the reference values were chosen to simulate process fluctuations caused, for example, by a contaminated protective window or incorrect laser power calibration. Four samples per setting were analyzed to obtain statistical meaningful results and obtain a measure for parameter set stability. Figure 27 shows the mechanical seam properties of the welded joints and Figure 28 shows the electrical contact resistance respectively.

It becomes evident that the circular beam oscillation shows the highest continuity in the mechanical and electrical properties when the laser power is varied. The linear stitched welds indicate slightly higher deviations in the measured data, which is attributed to the lower feed rates used for parameter sets #1 and #2. Thus, line energy differences are increased compared to welding strategies applying beam oscillation. In terms of absolute values, the linear stitched welds reveal the most appropriate values for a potential application for battery tab connections, as discussed earlier in Section 4.7. The vertical eight oscillation strategy is the most difficult to tune accurately to proper processing parameters, which leads to strongly differing values in Figure 25 and Figure 26, especially for parameter set #6. It is worth mentioning that for all parameter sets of the three welding strategies with higher line energy (#1, #3, and #5), a reduction in the laser power led to an improvement of the joint properties. This can be observed in the characteristic staircase-like progression in the evaluation results for parameter sets #1, #3, and #5 in Figure 25 and Figure 26. All investigated parameter sets for the three welding strategies can be found in Appendix B.

### 4.9. Discussion

In this study, the green laser wavelength with comparatively large spot diameter (340 um) was used to precisely control the element mixing in the fusion zone during laser beam welding copper and aluminum. The requirements for the welded joints include the highest possible tensile strength and low ohmic resistance in order to reduce heat losses in battery tab connections as far as possible. Due to the precise energy input at small keyhole aspect ratios with 515 nm wavelength (see [59]), overlap welds with suitable mechanical strength and electrical resistance values were successfully generated by the use of linear and oscillating welding strategies. Table 7 gives an overview of studies on dissimilar laser beam welding of copper and aluminum.

Compared to the results of other researchers, the current study based on the use of visible wavelength shows results to great advantage. Comparative mechanical strength and electrical resistance values were achieved in distinct processing parameter regions for the three different welding strategies compared to single mode near-infrared beam sources in pulsed or continuous-wave (cw) operation mode and a blue diode laser in [48]. The presented investigations quantified the influence of the processing parameters on the interface geometry and the resulting electrical and mechanical properties of the welds, as well as their influence on the amount and local distribution of intermetallic phases in the weld seams. In addition, joint repeatability was researched, identifying reproducible parameter sets for high volume battery tab joining. Furthermore, our study confirmed a correlation between the electrical resistance and the tensile strength of the joints. Since this relationship offers the possibility of non-destructive testing of the joint quality, which is much faster and cheaper than conventional tensile-shear strength tests, a special fixture with integrated sensor technology was developed, which is described in Section 4.10.

### 4.10. Clamping Device with Integrated Sensor Technology

**Figure 29:**
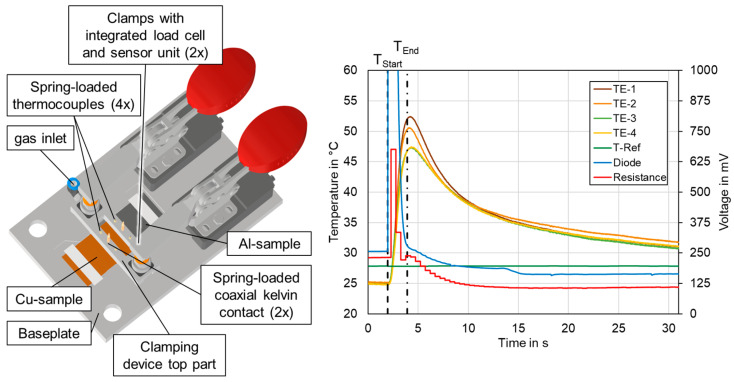
Sample fixture with integrated sensors for the in-line measurement of seam performance during welding.

The developed clamping device with integrated sensor technology for direct data acquisition before, during, and after the welding process close to the interaction zone is shown in Figure 29. The measurement results for a welding process using parameter set #2 are displayed in the diagram on the right.

The clamping device features the following structure and functionalities:

1. A 3D-printed clamping top part with integrated metal inserts in the specimen direction and in areas where clamping force is induced into the part.
2. An integrated gas inlet for supplying a protective gas (nitrogen) to the interaction zone of laser and material.
3. Two horizontal toggle clamps with integrated load cell and sensor unit (Kipp K1463, Heinrich Kipp Werk GmbH, Sulz am Neckar, Germany) for the measurement and assurance of the required clamping force before the welding process is started.
4. Two spring-loaded coaxial kelvin contacts (UWE electronic GmbH, Germany) with isolated inner and outer conductors for electrical resistance measurement (test length now 16 mm).
5. Four type K spring -loaded thermocouples (brass, gold plated; UWE electronic GmbH, Unterhaching, Germany) to determine the temperature increase in the samples with spatial resolution near the welded area (all sensors were integrated in holes in 8 mm distance to the symmetry plane of the weld seam).
6. A Si-photodiode PDA100A-EC (Thorlabs Inc., Newton, NJ, USA) mounted in the welding cabin and pointing to the laser interaction zone to sense the onset of laser emission for data recording using an Expert Key 200L (Delphin Technology AG, Bergisch Gladbach, Germany) data logger.

All thermocouples were connected to 18-bit A/D converted inputs of the data logger. The resistance measurement was triggered by the process start, while the signal was passed from the measurement device to the data logger by means of its analog output. The conversion factor was 100 mV = 0.1 mΩ. The clamping force was monitored before the start of the welding process to ensure proper contact (zero gap) of the joining partners. The rising edge of the photodiode signal at the start of the laser emission triggered the data recording, with 2 s recorded retrospectively. The signal drop at 13 s indicates the opening of the cabin door, as less light was reflected to the sensor and no filter was attached to the photodiode. From the signal trend (red), the decrease in electrical resistance due to the metallic bonding between copper and aluminum can be clearly seen. During the welding process however, no physically meaningful values can be extracted from the measurement setup for the electrical resistance. In addition, the rapid increase during the welding process (T_Start_ to T_End_) and the subsequent cooling can be read from the temperature measurements, which is already below 35 °C, 12 s after the end of laser emission. A difference in the cooling rate for the copper sheet on top compared to the aluminum sheet is also observed. The local temperature distribution reveals that the left half of the copper plate has a slightly higher maximum temperature than the right position (Positions: TE-1: Cu, seam start; TE-2: Cu, seam end; TE-3: Al, seam start; TE-4: Al, seam end, T-Ref: reference temperature data logger). This behavior may be related to the protective gas flow. Since the goal of the measurement chain is an accurate temporal resolution of the welding process, it enables process optimization with regard to the energy introduced into the components (temperature) and the seam quality (resistance). The methodology could also extend the easy determination of process stability once a suitable process parameter is identified. Further experiments are needed to investigate the potential in detail.

## 5. Conclusions

In this work, the seam properties and the process stability of different overlap welding strategies using green laser radiation for dissimilar copper to aluminum welding were investigated. The intermixing of both metals and phase formation were studied, as these connections are often encountered in the battery tab connections due to their good thermal and electrical conductivity. In this process, the intermixing is rapid because of the differences in density and melting temperatures of the joining partners and the deep penetration welding mode used. Thus, intermetallic phases may be formed, which are prone to reduce the performance of the weld seam. Therefore, the capabilities of applying green laser radiation in combination with and without spatial power modulation (linear stitched weld, circular and vertical eight beam oscillation) to affect the interconnection area of copper-aluminum joints were investigated. The most important contributions and findings of the present study can be summarized as follows.

- Process parameter windows were identified for all three welding strategies, with the linear stitched weld showing the most stable results. It is noticeable that there must be a sufficient intensity to enable stable incoupling and interface area between the components.
- The weld seam shape, characterized by penetration depth and seam width was reproducibly reached with copper as the top layer. The process windows are large compared to infrared laser applications of this type and the geometrical properties of the weld seams can be precisely controlled by the process parameters laser power and feed rate (v_F_, f for oscillating strategies).
- Using beam oscillation, a discontinuous weld seam is formed in the cross-sectional direction, which is a combination of different degrees of intermixing. Due to the overlapping oscillation path, the local energy changes compared to the linear stitched weld and copper aluminum intermixing in the interaction zone differs significantly.
- The results of the test series show a different sensitivity of the oscillating welding strategies with regard to the temperatures in the seam adjacent zone since heat accumulation effects cause higher maximum temperatures close to the permissible limit when using the vertical eight oscillation strategy. Overall, no critical temperatures were detected in the seam adjacent area in this study.
- The deep penetration mode used favors the formation of sound, crack-free weld seams. Pores were found to be distributed over the entire joint area when higher line energies were applied compared to the reference parameters.
- Based on the findings, the occurrence of intermetallic phases was investigated. EDS analysis confirmed that intermetallic phases were distributed in the interconnection area of copper and aluminum in the form of layers in the weld. Hardness measurements confirmed the presence of these intermetallic phases (plausibly θ and η). In the case of the oscillating welding strategies, these were distributed over the entire fusion zone, while in the case of the linear stitched weld, a concentration was observed at the interface.
- Excellent mechanical properties were observed for the reference welding parameter sets defined for of all welding strategies, with tensile strengths similar to those obtained in the literature with near-infrared single-mode laser welding.
- A maximum mechanical resistance of 880 N was found. The failure of the joints was classified based on the fracture location. The weld seams were found to fail outside the fusion zone despite the presence of brittle intermetallic phases in the joint. A large plastic deformation of the aluminum sheet after fracture indicates a ductile weld seam behavior. Only for increased intermixing using the vertical eight beam oscillation, a fracture in the weld seam occurred.
- Based on the microstructural analysis and tensile-shear strength testing, it can be concluded that maximum shear strength is achieved in copper-aluminum overlap welds with low degree of intermixing and without the presence of large complex intermetallic structures in the weld seam.
- The electrical resistance is observed to be relatively stable and not significantly sensitive to process parameters. The measured values were comparable to those reported in the literature. The linear stitched welded samples performed slightly better, and a lower deviation was detected, with the lowest value of 42 μΩ. In addition, a correlation is found between the electrical resistance and the mechanical strength of the weld.
- Finally, a clamping device with integrated sensor technology (clamping force, resistance, temperature) for direct data acquisition before, during, and after the welding process close to the interaction zone was developed and tested.

In summary, this paper demonstrated the high feasibility of producing battery tab connections by laser beam welding using green laser radiation with appropriate seam properties. These in-depth analyses of weld seam properties, intermetallic phases, electrical contact resistance, and temperature rise should enable the development of an efficient battery system that meets critical quality requirements.

In future work, the long-term stability of this connections with regard to cyclic loads induced by temperature or current may be investigated. An experimental study correlating the electrical resistance with the thickness of the intermetallic layer in the weld seam is also of interest and proposed for future studies.

## Figures and Tables

**Table 1:** Intermetallic phases of copper and aluminum and their physical properties [31,32].

Phase	Chemical Composition	Cu Mass in %	Al Mass in %	Hardness in HV	Specific Resistivity in µΩ cm	ΔG in kJ mol^−1^
Cu	Cu	100	0	100	1.8	-
γ_1_	Al_4_Cu_9_	80	20	1050	14.2	−21.69
δ	Al_2_Cu_3_	78	22	180	13.4	−20.67
ζ_2_	Al_3_Cu_4_	75	25	624	12.2	−20.64
η_2_	AlCu	70	30	648	11.4	−19.92
Θ	Al_2_Cu	55	45	413	8.0	−13.05
Al	Al	0	100	60	2.9	-

**Table 2:** Laser beam source and optical setup used for the investigation.

Dimension	Unit	Trumpf TruDisk 3022
Wavelength (λ)	[nm]	515
Laser power (P_max_)	[W]	3000
Fiber diameter (d_LLK_)	[µm]	200
Focal length collimator (f_C_)	[mm]	150
Focal length optics (f_F_)	[mm]	255
Focal diameter (d_F_, measured)	[µm]	342.5
Diffraction factor (M^2^)	[-]	46.1
Divergence angle (θ)	[mrad]	88.2
Beam parameter product (BPP)	[mm∙mrad]	7.55

**Table 3:** Chemical composition of AlN30 and Ni-plated copper in wt. %.

Material/Element	Al	Zn	Mg	Mn	Cu	Si + Fe
Al (AlN30)	99.42	0.01	0.01	0.01	0.01	0.47
Cu (Cu, 2.5 µm Ni-layer)	-	-	-	-	99.96	-

**Table 4:** Process parameters developed from the preliminary study for different overlap welding strategies from copper to aluminum.

Identification	Welding Strategy	P_L_ in W	v_F_ in mm/s	f in Hz
#1	Linear	1400	400	-
#2	Linear	1600	500	-
#3	O-oscillation	900	50	50
#4	O-oscillation	1500	150	150
#5	8-oscillation	800	30	75
#6	8-oscillation	800	25	100

**Table 5:** Compilation of metallographically approved process parameters developed for different overlap welding strategies from copper to aluminum (repetition and extension of Table 4).

Identification	Welding Strategy	P_L_ in W	v_F_ in mm/s	f in Hz	$\bar{v_{p}}$ in mm/s	E_In_ in J	l_Trajectory_ in mm	Reference
#1	Linear	1400	400	-	400	11	99	Figure 5c
#2	Linear	1600	500	-	500	10	99	Figure 5g
#3	O-oscillation	900	50	50	236	582	153	Figure 7c
#4	O-oscillation	1500	150	150	707	323	153	Figure 7g
#5	8-oscillation	800	30	75	530	838	753	Figure 9c
#6	8-oscillation	800	25	100	707	990	565	Figure 9b

**Table 6:** Hardness measurements of the base material.

Identification	Hardness in HV (HV 0.1)
Copper	58.1 ± 2.9
Aluminum	22.6 ± 1.6

**Table 7:** Summary of research conducted on laser beam welding of copper and aluminum.

Materials	Weld Length	Laser Process	Joint Type	Welding Strategy	Maximum Load	Electrical Contact Resistance	Reference (Year)
0.2 mm Cu [Ni]/0.3 mm Al	32 mm	Yb:YAG (2f) λ = 515 nm (cw)	Lap joint (Cu on top)	Linear Oscillating	880 N	42 μΩ (Test length 17 mm)	(this work) 2023
0.2 mm Al/1 mm Cu	45 mm	Single-mode fiber (pulsed mode)	Lap joint (Al on top)	Oscillating	1209 N	86 μΩ (Test length 40 mm)	[71] 2023
0.3 mm Cu/0.4 mm Al	45 mm	Diode λ = 450 nm (cw)	Lap joint (Cu on top)	Linear	~670 N	44 μΩ (Test length 20 mm)	[48] 2022
0.3 mm Cu [Ni]/0.45 mm Al	45 mm	Single-mode fiber (cw)	Lap joint (Cu on top)	Linear	700–800 N	40~42 μΩ (Test length 20 mm)	[73] 2022
0.4 mm Al/0.3 mm Cu	45 mm	Single-mode fiber (pulsed mode)	Lap joint (Al on top)	Linear	~107 kg (1049 N)	N/A	[74] 2019
0.3 mm Cu/0.4 mm Al	45 mm	Single-mode fiber (cw)	Lap joint (Cu or Al on top)	Linear Oscillating	~130 kgf (1274 N)	N/A	[75] 2019
0.3 mm Cu [Ni]/0.45 mm Al	45 mm	Single-mode fiber (cw)	Lap joint (Cu or Al on top)	Oscillating	~120 kgf (1177 N)	Low electrical resistance	[43] 2019
0.3 mm Cu/0.3 mm Al	20 mm	Nd:YAG (cw)	Lap joint (Cu on top)	Linear	539.52 N	N/A	[67] 2014

## Data Availability

Not applicable.

## Appendix A

The overlap degree U for a circular beam oscillation with the linear feed rate $\;v_{f}$, the oscillation frequency f, the oscillation amplitude a, and the time t can be calculated by use of Equation (A1).

(A1) $$U=\frac{\sqrt{4\cdot a^{2}-{\left(\frac{v_{f}}{\pi \cdot f}\right)}^{2}}-\frac{v_{f}}{2\cdot \pi \cdot f}\cdot \left[3\cdot \pi +2\cdot \arcsin\left(-\frac{v_{f}}{2\cdot f\cdot a}\right)\right]}{\sqrt{4\cdot a^{2}-{\left(\frac{v_{f}}{\pi \cdot f}\right)}^{2}}-\frac{v_{f}}{2\cdot \pi \cdot f}\cdot \left[\pi +2\cdot \arcsin\left(-\frac{v_{f}}{2\cdot f\cdot a}\right)\right]}\cdot 100\%$$

## Appendix B

**Table A1:** Examined parameter sets for the welding strategies circular beam oscillation (left and center) and vertical eight beam oscillation (right); reference parameter sets marked in green.

P_L_ in W	v_F_ in mm/s	f in Hz		P_L_ in W	v_F_ in mm/s	f in Hz		P_L_ in W	v_F_ in mm/s	f in Hz
400	30	50		800	75	150		700	25	100
500	30	50		900	75	150		800	25	100
600	30	50		950	75	150		850	25	100
700	30	50		1000	75	150		900	25	100
800	30	50		1100	75	150		600	30	50
600	30	75		850	75	200		700	30	50
700	30	75		900	75	200		600	30	75
800	30	75		950	75	200		700	30	75
600	30	100		1000	75	200		800	30	75
700	30	100		800	100	100		900	30	75
800	30	100		900	100	100		800	50	50
600	50	50		1000	100	100		900	50	50
700	50	50		1100	100	100		1000	50	50
800	50	50		1200	100	100		1100	50	50
900	50	50		1100	100	150		900	50	100
700	50	75		1200	100	150		950	50	100
800	50	75		1300	100	150		1000	50	100
900	50	75		800	100	200		1100	50	100
1000		75		900	100	200		1000	75	100
700	50	100		1000	100	200		1100	75	100
800	50	100		1100	100	200		1200	75	100
900	50	100		1200	100	200		800	75	125
1000	50	100		1200	150	150		900	75	125
700	50	150		1300	150	150		1000	75	125
800	50	150		1400	150	150		900	100	100
900	50	150		1500	150	150		1000	100	100
1000	50	150		1600	150	150		1100	100	100
800	75	100		800	150	200		1200	100	100
900	75	100		1000	150	200		800	100	125
1000	75	100		1400	150	200		900	100	125
1100	75	100		1600	150	200		1000	100	125
1200	75	100		1800	150	200		1100	100	125
								1200	100	125
								900	125	125
								1000	125	125
								1100	125	125
								1200	125	125
								1300	125	125

**Table A2:** Examined parameter sets for the linear stitched weld; reference parameter sets marked in green.

P_L_ in W	v_F_ in mm/s
1200	300
1300	300
1400	300
1500	300
1300	400
1400	400
1500	400
1600	400
1500	500
1600	500
1700	500
1800	500
